# Cerebrovascular Effects of Sildenafil in Small Vessel Disease: The OxHARP Trial

**DOI:** 10.1161/CIRCRESAHA.124.324327

**Published:** 2024-06-04

**Authors:** Alastair J.S. Webb, Jacqueline S. Birks, Karolina A. Feakins, Amy Lawson, Jesse Dawson, Alexander M.K. Rothman, David J. Werring, Osian Llwyd, Catriona R. Stewart, James Thomas

**Affiliations:** Wolfson Centre for Prevention of Stroke and Dementia (A.J.S.W., K.A.F., A.L., O.L., C.R.S., J.T.), University of Oxford, United Kingdom.; Centre for Statistics in Medicine, Botnar Research Centre (J.S.B.), University of Oxford, United Kingdom.; Department of Brain Sciences, Imperial College London, United Kingdom (A.J.S.W.).; School of Cardiovascular and Metabolic Health, University of Glasgow, United Kingdom (J.D.).; Department of Cardiovascular Science, University of Sheffield, United Kingdom (A.M.K.R.).; Research Department of Brain Repair and Rehabilitation, Institute of Neurology, University College London, United Kingdom (D.J.W.).

**Keywords:** cerebral small vessel diseases, cilostazol, perfusion, white matter

## Abstract

**BACKGROUND:**

Vascular cognitive impairment due to cerebral small vessel disease is associated with cerebral pulsatility, white matter hypoperfusion, and reduced cerebrovascular reactivity (CVR), and is potentially improved by endothelium-targeted drugs such as cilostazol. Whether sildenafil, a phosphodiesterase-5 inhibitor, improves cerebrovascular dysfunction is unknown.

**METHODS:**

OxHARP trial (Oxford Haemodynamic Adaptation to Reduce Pulsatility) was a double-blind, randomized, placebo-controlled, 3-way crossover trial after nonembolic cerebrovascular events with mild-moderate white matter hyperintensities (WMH), the most prevalent manifestation of cerebral small vessel disease. The primary outcome assessed the superiority of 3 weeks of sildenafil 50 mg thrice daily versus placebo (mixed-effect linear models) on middle cerebral artery pulsatility, derived from peak systolic and end-diastolic velocities (transcranial ultrasound), with noninferiority to cilostazol 100 mg twice daily. Secondary end points included the following: cerebrovascular reactivity during inhalation of air, 4% and 6% CO_2_ on transcranial ultrasound (transcranial ultrasound-CVR); blood oxygen-level dependent–magnetic resonance imaging within WMH (CVR-WMH) and normal-appearing white matter (CVR-normal-appearing white matter); cerebral perfusion by arterial spin labeling (magnetic resonance imaging pseudocontinuous arterial spin labeling); and resistance by cerebrovascular conductance. Adverse effects were compared by Cochran Q.

**RESULTS:**

In 65/75 (87%) patients (median, 70 years;79% male) with valid primary outcome data, cerebral pulsatility was unchanged on sildenafil versus placebo (0.02, −0.01 to 0.05; *P*=0.18), or versus cilostazol (−0.01, −0.04 to 0.02; *P*=0.36), despite increased blood flow (∆ peak systolic velocity, 6.3 cm/s, 3.5–9.07; *P*<0.001; ∆ end-diastolic velocity, 1.98, 0.66–3.29; *P*=0.004). Secondary outcomes improved on sildenafil versus placebo for CVR-transcranial ultrasound (0.83 cm/s per mm Hg, 0.23–1.42; *P*=0.007), CVR-WMH (0.07, 0–0.14; *P*=0.043), CVR-normal-appearing white matter (0.06, 0.00–0.12; *P*=0.048), perfusion (WMH: 1.82 mL/100 g per minute, 0.5–3.15; *P*=0.008; and normal-appearing white matter, 2.12, 0.66–3.6; *P*=0.006) and cerebrovascular resistance (sildenafil-placebo: 0.08, 0.05–0.10; *P*=4.9×10^−8^; cilostazol-placebo, 0.06, 0.03–0.09; *P*=5.1×10^−5^). Both drugs increased headaches (*P*=1.1×10^−4^), while cilostazol increased moderate-severe diarrhea (*P*=0.013).

**CONCLUSIONS:**

Sildenafil did not reduce pulsatility but increased cerebrovascular reactivity and perfusion. Sildenafil merits further study to determine whether it prevents the clinical sequelae of small vessel disease.

**REGISTRATION:**

URL: https://www.clinicaltrials.gov/study/NCT03855332; Unique identifier: NCT03855332.

**Novelty and Significance**

**What Is Known?**

- Cerebral small vessel disease is associated with 30% of ischemic stroke, 80% of hemorrhagic stroke, and ≈40% of dementia but has no specific treatment.
- Small vessel disease is associated with cerebral pulsatility, white matter hypoperfusion and reduced cerebrovascular reactivity, and these mechanisms, and associated clinical outcomes, may be improved by drugs targeting the endothelium, such as isosorbide mononitrate and cilostazol.
- Sildenafil, a phosphodiesterase-5 inhibitor, is an effective systemic vasodilator that targets endothelial function, but its cerebrovascular effects and potential in small vessel disease is unknown.

**What New Information Does This Article Contribute?**

- In 75 patients with symptomatic cerebral small vessel disease, sildenafil did not reduce cerebral pulsatility compared with placebo but improved cerebrovascular reactivity, cerebrovascular resistance, and cerebral blood flow.
- This is the first study to demonstrate that targeting endothelial function in small vessel disease improves both cerebral blood flow and dynamic cerebrovascular function by combining transcranial ultrasound and magnetic resonance imaging.
- Sildenafil was noninferior to cilostazol and associated with less side effects.

Cerebral small vessel disease is responsible for a large population burden of disease due to stroke and cognitive decline but has no specific treatment. Drugs that target endothelial function have shown potential benefit in preventing cognitive decline. Sildenafil (commonly known as Viagra) is a well-tolerated, widely used vasodilator but whether it has potentially beneficial cerebrovascular effects is unknown. OxHARP trial (Oxford Haemodynamic Adaptation to Reduce Pulsatility Trial) was a classical, double-blind crossover trial where 75 participants with mild-moderate small vessel disease and a prior minor stroke received placebo, sildenafil and cilostazol in randomized order for 3 weeks each, with at least a 1-week washout. Although sildenafil did not reduce cerebral pulsatility on transcranial ultrasound compared with placebo (the primary outcome), it did improve endothelial function measured by cerebrovascular reactivity on ultrasound and magnetic resonance imaging, and improved cerebrovascular resistance and perfusion. It was also noninferior to cilostazol which did not significantly increase cerebrovascular reactivity compared with placebo and caused less diarrhea. OxHARP, therefore, demonstrated the potential of a new paradigm including detailed physiological testing and combining ultrasound and magnetic resonance imaging to identify drugs with potential benefits in small vessel disease. Furthermore, it demonstrated that sildenafil improves cerebrovascular dysfunction and is a promising candidate for further clinical trials.

**Meet the First Author, see p 264**

**Editorial, see p 332**

Cerebral small vessel disease (cSVD) is due to chronic damage to the small vessels of the brain. It is evident on magnetic resonance imaging (MRI) in over half of people over 65^1^ as dilated perivascular spaces, lacunar strokes, microbleeds, or white matter hyperintensities (WMHs), with WMH being the most prevalent manifestation of cSVD responsible for significant morbidity.^2^ It causes up to 30% of ischemic strokes, 80% of hemorrhagic strokes,^3^ and 40% of all-cause dementia, but there is no specific treatment^4^ due to a limited understanding of the pathophysiology of cSVD^5,6^ and few clinical trials.^7^ cSVD is strongly associated with midlife hypertension and its long-term consequences,^8^ including reduced white matter perfusion^9^; increased arterial stiffness^10^; and resulting increased cerebral arterial pulsatility.^11^ Pulsatility is particularly strongly associated with cSVD,^12^ is highly reproducible and parallels disease progression.^13^ cSVD is also strongly associated with endothelial dysfunction, manifested as blood-brain barrier breakdown^11^ and reduced cerebrovascular reactivity (CVR).^5^ These modifiable physiological outcomes provide short-term targets to support translational mechanistic trials.^14^

Drugs targeting endothelium-dependent vasodilatation are leading candidates to reduce harm in cSVD. Isosorbide mononitrate (ISMN) improved CVR in a 26-patient pilot trial,^15^ and improved cognitive outcomes in 363 patients in the LACI 2 trial (Lacunar Intervention-2) feasibility trial.^16^ The PDE3i (phosphodiesterase 3 inhibitor) cilostazol reduced cerebral pulsatility 90 days after lacunar stroke^17^ and reduced recurrent stroke risk in patients with cSVD within multiple large randomized controlled trials (RCTs).^18^ In contrast, the recent TREAT-SVDs trial (Effect of Amlodipine and Other Blood Pressure Lowering Agents on Microvascular Function in Small Vessel Diseases) found no difference in the physiological effects of amlodipine versus atenolol or losartan on CVR in sporadic cSVD.^19^ However, mechanistic trials in cSVD have largely assessed single physiological outcomes, limiting understanding of their physiological effects.

PDE5is (phosphodiesterase-5 inhibitors) increase smooth muscle cGMP in response to endothelial NO release^20^ and improve endothelial dysfunction in erectile dysfunction, pulmonary arterial hypertension, and Raynaud disease. By enhancing the smooth muscle response to endothelial NO, they are predicted to improve blood flow and increase CVR, while vasodilatation could reduce blood pressure augmentation,^21,22^ improve dampening of the arterial waveform, and thus reduce pulsatility. They have been associated with reduced dementia in large populations^23^ and amyloid-independent benefits in animal models,^24^ but there are no mechanistic studies of their sustained hemodynamic effect in cSVD.^25,26^ However, given the widespread use of PDE5i, their excellent tolerability, minimal interactions with established preventative treatments and a biologically plausible mechanism, they are good candidates to reduce the clinical sequelae of cSVD.

The OxHARP trial (Oxford Haemodynamic Adaptation to Reduce Pulsatility) randomized, double-blind, placebo-controlled crossover clinical trial established a physiological paradigm to test the effects of sildenafil on cerebral arterial pulsatility and CVR in patients with symptomatic mild to moderate small vessel disease, testing superiority compared with placebo and noninferiority to cilostazol.

## METHODS

### Data Availability

The data that support the findings of this study are available from the corresponding author upon reasonable request. Proposals should be directed to alastair.webb@ndcn.ox.ac.uk; to gain access, data requestors will need to sign a data access agreement.

### Study Design

OxHARP was a double-blind, randomized, placebo-controlled, 3-way crossover phase 2 trial with physiological end points,^27^ which ran from July 11, 2019 to the last visit of the last patient on December 6, 2022, with a 6-month pause due to the COVID-19 pandemic from March to September 2020. All procedures were performed at the Wolfson Center for Prevention of Stroke and Dementia. OxHARP is sponsored by the University of Oxford, approved by the UK Health Research Authority and South Central, Oxford C Research Ethics Committee (19/SC/0022), and is registered with https://www.clinicaltrials.gov (NCT03855332). The study protocol has been published previously.^27^

### Participants

OxHARP aimed to include 75 participants with a previous cryptogenic or lacunar stroke or TIA requiring secondary preventative treatment, with mild to moderate WMH evident on their most recent clinical brain imaging within the past 6 years (Fazekas score on MRI or modified Blennow score on CT of 1–3 <60 or 1–4 >60), allowing for 15% drop out. Inclusion and exclusion criteria are provided in Supplemental Material. Following referral from stroke services or identification via a research registry, potential participants were screened, provided face-to-face written consent, and gave demographic, clinical, and cognitive data. Transcranial ultrasound was performed at screening to confirm adequate temporal bone windows for measurement of cerebral pulsatility index (PI). The full inclusion criteria have been reported previously.^27^ All participants without a contraindication to MRI were consecutively invited to join the MRI substudy. This initially aimed to recruit 30 participants to be scanned on sildenafil and placebo but the trial was amended to recruit up to a further 30 participants to undergo MRI on all 3 treatments in May 2020. Participants who did not tolerate MRI were eligible to continue with transcranial ultrasound (TCD) alone.

### Randomization

The sequence of drug treatments was randomly allocated pretreatment by study number by the manufacturing pharmacy, stratified by MRI substudy. Medications were dispensed by the clinical trial pharmacy independently of the blinded study team, in scheduled treatment packs containing over-encapsulated, identical medications. All participants, researchers, and study physicians were blinded to drug allocation. To assess unblinding, men were asked about change in sexual activity and tumescence. The drug allocation code was held by the dispensing trial pharmacy in case of serious adverse events requiring medical intervention.

### Procedures

Potential participants underwent a telephone screening followed by a face-to-face screening visit at least 1 week later. The baseline assessment was completed either at screening or within 1 month. A standardized demographic, clinical and medication history, and clinical examination were performed by a study physician, including baseline cognitive testing (Montreal Cognitive Assessment, digit-symbol coding task, and fluid intelligence task), blood tests, and ECG. At baseline and all follow-up visits, a standardized physiological assessment was performed. MRI was performed at follow-up visits in the MRI substudy.

Following each visit, participants received treatment in randomized order with overencapsulated, double-blind medication. Each treatment lasted for 3 weeks, starting with either thrice-daily placebo, thrice-daily sildenafil 25 mg, or twice daily cilostazol 50 mg with a placebo dose at midday. After 1 week, the dose was doubled by taking 2 tablets at each dose, unless limited by side effects. There was a minimum 1-week wash out between drugs. Assessments were performed on the final day of treatment.

Physiological assessments were performed in a temperature-controlled laboratory (21–23°C) after 20-minute supine rest, timed to be 30 minutes after observed administration of trial medication in clinic. Middle cerebral artery (MCA) flow velocity was assessed by transcranial ultrasound via the transtemporal window to derive the primary outcome: Gosling pulsatility index ([peak systolic velocity–end-diastolic velocity]/mean flow velocity). The principal secondary outcome was CVR, assessed during bilateral TCD monitoring of the MCA with concurrent ECG, noninvasive blood pressure monitoring calibrated to an oscillometric brachial reading (FMS, Finometer Midi), and end-tidal carbon dioxide monitoring (etCO_2_, AD Instruments Gas Analyser ML206). After 10 minutes rest, CVR was assessed during 2-minute alternating periods of inhalation of medical air, 4% CO_2_ and 6% CO_2_, delivered via a respiratory circuit with a well-sealed, noninvasive ventilation mask. Aortic blood pressure was determined by radial artery applanation tonometry (Sphygmocor).^27^

After completion of physiological testing, participants in the MRI substudy underwent structural MRI including T1, fluid attenuated inversion recovery (FLAIR), susceptibility-weighted imaging, and diffusion tensor imaging, distributed between the first 2 MRI visits. CVR on MRI was assessed at each MRI follow-up on multiband blood oxygen-level–dependent MRI with whole brain acquisition every 800 ms, with perfusion imaging with pseudocontinuous arterial spin labeling. Details of the imaging protocol have been reported previously.^27^ During CVR, participants breathed medical air or 6% CO_2_ (balance medical air) in 2 sets of 2-minute alternating periods, delivered by the same respiratory circuit used during CVR assessment with TCD. Throughout imaging, participants had continuous noninvasive monitoring of etCO_2_ (AD Instruments Gas Analyser ML206), respiratory motion, oxygen saturations, and continuous blood pressure.

### Outcomes

The primary outcome was Gosling MCA-PI ([peak systolic velocitiy–end-diastolic velocity]/mean flow velocity) on transcranial ultrasound, derived from the average of 3 manually measured peak systolic and 3 end-diastolic velocities, from each of 2 recordings, reviewed, and quality assessed by 2 blinded reviewers (A.J.S.W. and J.T.). Where both MCA-PI recordings were of similar quality, with no measurement artifacts and a similar mean velocity (within 10%), an average was taken. Otherwise, the higher quality recording was used. Disagreements between reviewers were resolved by a panel discussion. The secondary outcome was CVR on TCD, with the optimal side selected by the same process as for MCA-PI, with 2 blinded reviewers (A.J.S.W. and O.L.). This was defined as change in mean flow velocity per mm Hg change in etCO_2_ from the β-coefficient from a linear model between the etCO_2_ value during inhalation of medical air, 4% and 6% CO_2_ and mean MCA flow velocity, after correction for phase delay by cross-correlation with piecewise cubic Hermitte interpolation. Tertiary outcomes included effects of each drug on aortic blood pressure, on the physiological measurements used to derive the primary and secondary outcomes (peak systolic velocitiy, end-diastolic velocity, and mean cerebral blood flow velocity) and on the relationship between them as a measure of cerebrovascular resistance, estimated as the cerebrovascular conductance index (cerebrovascular conductance index=mean cerebral blood flow velocity/aortic mean blood pressure [MBP]).

Blood oxygen-level–dependent-CVR images were preprocessed (Motion Correction with fMRIBs Linear Registration Tool [MCFLIRT], B0 unwarping Boundary Based Registration [BBR], high-pass temporally filtered at 300 s and brain extracted). Regions of interest were defined conservatively by segmentation of T1 images by tissue type (fMRIB Software Library [FSL]: FAST [fMRIB automated segmentation tool]/FIRST), erosion by 1 voxel and segmentation into white matter hyperintensities (WMH) and normal-appearing white matter (FSL:BIANCA), followed by registration to structural and standard space (FLIRT and FNIRT). Phase delays between recorded etCO_2_ and each voxel time series were estimated by cross-correlation to the maximum r^2^ (Matlab, in-house software) and phase-shifted by piecewise cubic Hermitte interpolation. Associations between phase-shifted etCO_2_ time series and each voxel are determined by general linear models on a voxel-wise basis (Matlab) and by FEAT (FMRI Expert Analysis Tool), expressing CVR as the average percentage change in blood oxygen-level–dependent response per mm Hg change in etCO_2_ across all voxels in the regions of interest (normal-appearing white matter and WMH). In a sensitivity analysis, the average voxel-wise, within-individual difference for each MRI outcome is determined following registration to standard space. WMH volume on MRI was quantified for OxHARP trial scans from FLAIR images (BIANCA, FSL).

Safety and adverse events were assessed face-to-face by a study physician at each visit, including standardized assessment of the most frequent adverse events (headache, flushing, edema, breathlessness, lightheadedness, visual disturbance, bruising/bleeding, diarrhea, and priapism). All adverse events were reviewed by the study chief investigator (A.W.) for assignment of severity and causality, before unblinding. SAEs were reported to the independent Data Safety Monitoring Board (DSMB) within 24 hours, which met every 6 months for a blinded review of recruitment and adverse events rates, with unblinded data review if requested.

### Statistical Analysis

The primary drug comparisons used mixed-effect linear models, adjusted for age, sex, visit order, and allocation randomization sequence, with the primary study outcome defined as the difference between sildenafil versus placebo on cerebral pulsatility (MCA-PI). Secondary outcomes included CVR on TCD and CVR in WMH and normal-appearing white matter on MRI. Noninferiority of sildenafil versus cilostazol on MCA-PI was assessed by the lower margin of the CI compared with the predefined noninferiority threshold (0.08 for MCA-PI). Cilostazol was compared with placebo as for the primary outcome. The primary analysis used mixed-effect linear models to allow for missing data due to medication side effects and increased time intervals due to COVID. Mediation analysis was used to assess whether drug effects on cerebral transcranial ultrasound indices were mediated by effects on aortic blood pressure, and whether CVR on MRI was mediated by effects on the MCA. Rates of adverse events were compared across all treatments in participants receiving all 3 treatments by Cochran Q, with post hoc pairwise comparisons by McNemar test. All analyses were performed in R, Matlab or Stata. The protocol and statistical analysis plan have been published previously. The funder of the study had no role in study design, data collection, data analysis, data interpretation, or writing of the report.

## RESULTS

**Figure 1.**
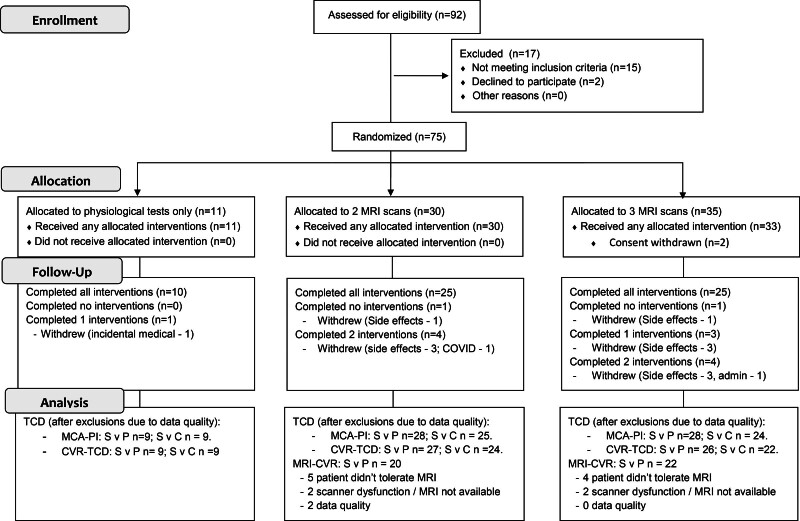
**Consort flow diagram.** CVR-TCD indicates cerebrovascular reactivity; MRI, magnetic resonance imaging; and MCA-PI, middle cerebral artery pulsatility index.

**Figure 2.**
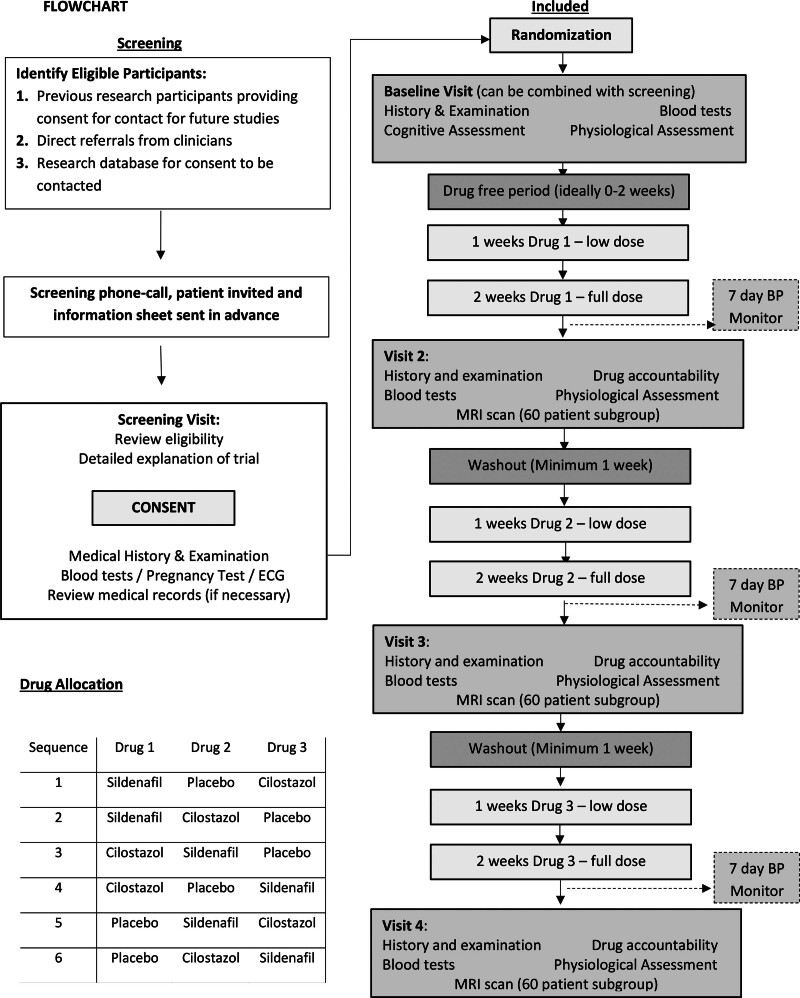
**Study flow diagram.** BP indicates blood pressure; and MRI, magnetic resonance imaging. *Note that participants will only go up to the full dose if they are not on omeprazole.

Of 92 potential participants screened face-to-face, 17 participants were not eligible due to comorbidities (Figure 1), while 2 randomized participants withdrew before receiving any medication. Thirty-five participants consented to 3 MRIs, 30 to 2 MRIs, and 10 participants only to TCD assessment. Sixty-five (89%) participants had primary outcome data, 63 (86%) had valid data for the CVR comparison by TCD and 43 (59%) had valid data for sildenafil versus placebo on MRI (Figures 1 and 2). The OxHARP population had a median age of 70, the majority were men (78%) and had a previous stroke (60%), with nearly equal participants with mild (53%) versus moderate (26%) or moderately severe (20%) white matter hyperintensities (Table 1). Participants opting only to undergo physiological testing were slightly older, with lower CVR and cerebral blood flow velocities.

**Table 1.**
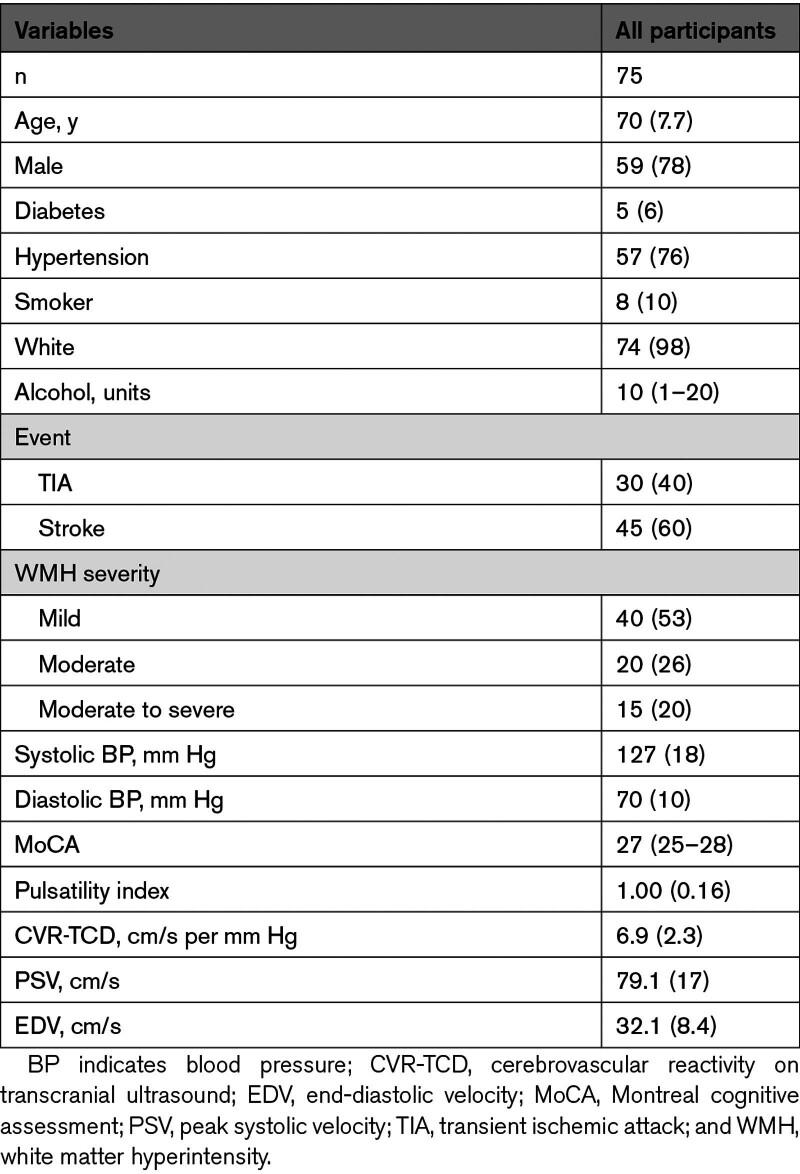
Clinical Characteristics of Population Included in the Oxford Haemodynamic Adaptation to Reduce Pulsatility Trial

**Figure 3.**
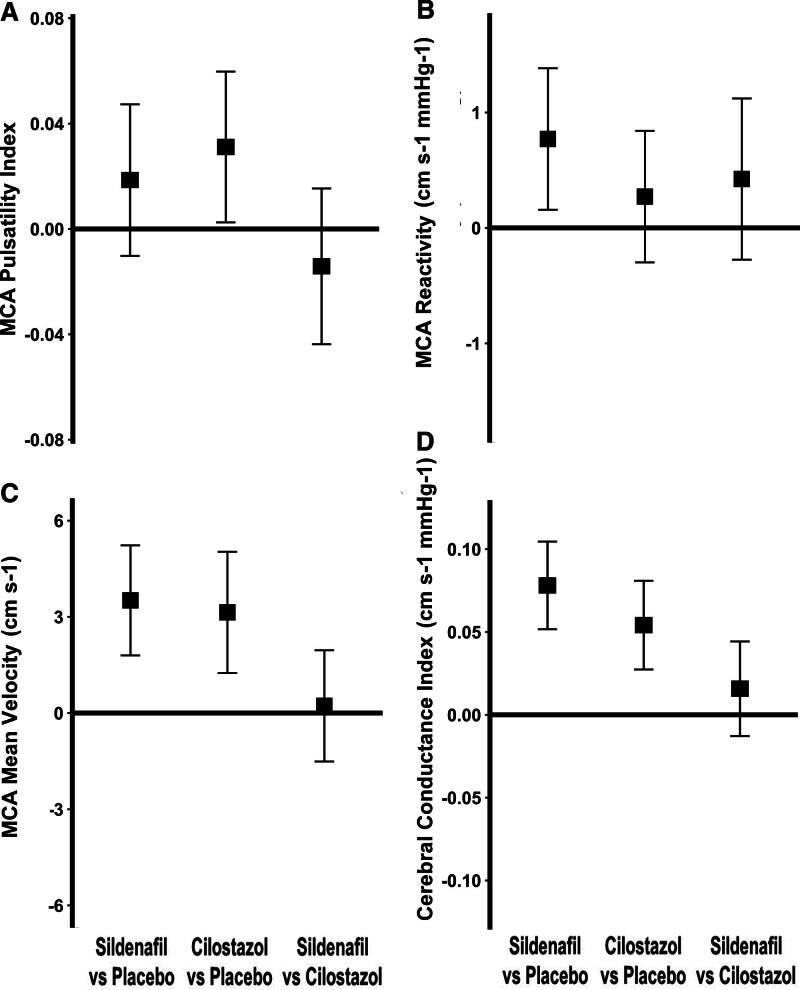
**Differences between sildenafil, cilostazol, and placebo on middle cerebral artery (MCA) blood flow, pulsatility, and reactivity on transcranial ultrasound.** The mean, within-individual difference for the first drug vs the second is shown with 95% CIs for the following: **A**, MCA pulsatility index (MCA-PI). **B**, Cerebrovascular reactivity from the β-coefficient from a linear model for the association between end-tidal CO_2_ and mean flow velocity (cerebrovascular reactivity [CVR]). **C**, Mean MCA flow velocity (MFV). **D**, Cerebrovascular conductance index (CVCi−MFV/aortic mean blood pressure [MBP]). Differences are shown for sildenafil minus placebo (S-P), sildenafil minus cilostazol (S-C), and cilostazol minus placebo (C-P).

There was no significant difference between the effect of sildenafil and placebo on MCA-PI (Table 2; Figure 3), including when analyzed as difference from baseline. Sildenafil was noninferior to cilostazol (upper CI=0.02 compared with the noninferiority threshold of 0.08), but there was a significant increase in MCA-PI on cilostazol versus placebo (Table 2; Figure 3). There was no evidence of a carry over effect with no association or interaction with randomization order or visit number.

**Table 2.**
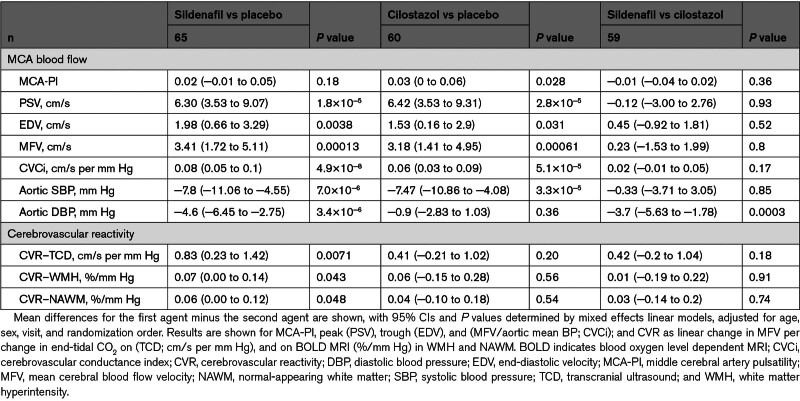
Differences Between Sildenafil, Placebo, and Cilostazol on Cerebral Pulsatility, Cerebrovascular Reactivity, and Associated Physiological Measures

**Figure 4.**
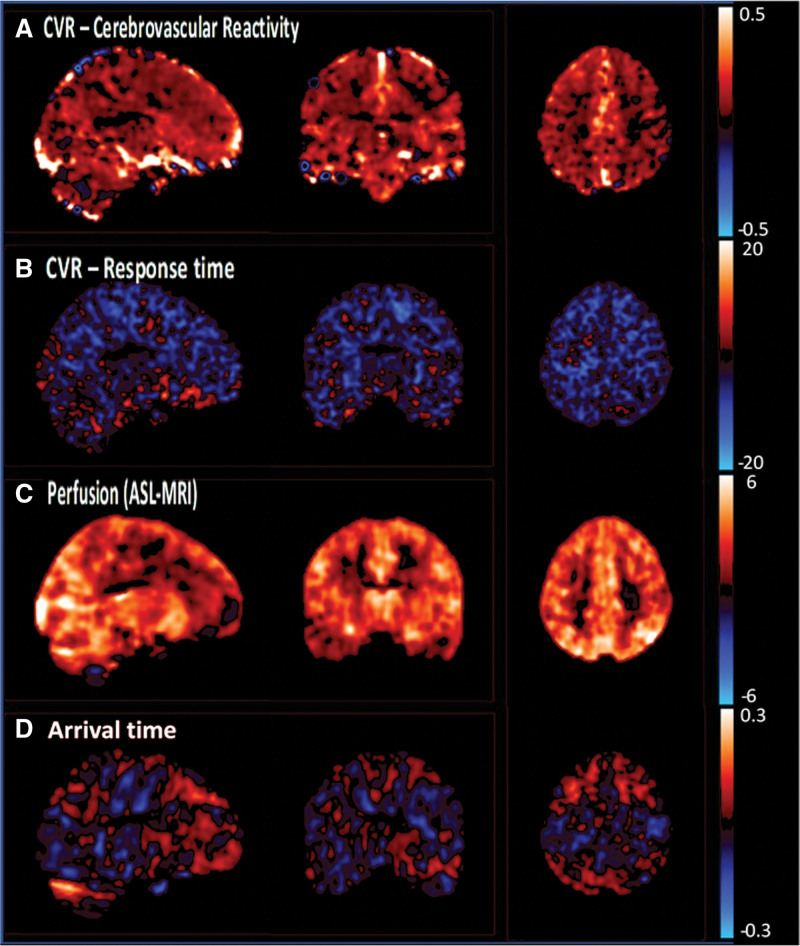
**Differences between sildenafil and placebo on cerebrovascular reactivity (CVR) and cerebral blood flow on magnetic resonance imaging (MRI).** The mean, within-individual difference on MRI for sildenafil minus placebo is shown on a voxel-wise basis, following registration to standard MNI space and Gaussian smoothing. Results are shown as follows. **A**, CVR as percentage change in BOLD per mm Hg end-tidal CO_2_. **B**, CVR response: difference in time delay between end-tidal carbon dioxide monitoring and BOLD signal in seconds. **C**, Difference in perfusion on ASL, in milliliter per minutes per 100 g. **D**, Difference in arrival time of blood flow during ASL, in seconds. ASL indicates arterial spin labelling; BOLD, blood oxygen level dependent imaging; and MNI, Montreal Neurological Institute.

Sildenafil significantly increased TCD-CVR in the MCA compared with placebo (*P*=0.0071; Table 2; Figure 3). This was consistent with increased CVR on sildenafil versus placebo on MRI within white matter hyperintensities and normal-appearing white matter, after adjustment for confounders (Figure 4; Tables S3 and S4), although with only a trend to increased CVR in unadjusted comparisons (Table S3). Effects on parenchymal reactivity on MRI may have been partially mediated by effects on the middle cerebral artery, but with only borderline significance (Table S4). There was no significant difference between sildenafil and cilostazol on TCD-CVR (+0.47; *P*=0.14). In addition to the increased magnitude of CVR with sildenafil, there was a more rapid CVR response to carbon dioxide with sildenafil versus placebo (Figure 2; Table S4). There was no difference between cilostazol with either placebo or sildenafil on MRI (Tables S3 and S4; Figures S4 and S5), but the study was underpowered for the comparison of cilostazol and placebo on MRI, with only 15 participants having MRI scans on both. In other tissues, CVR was greater with sildenafil than placebo in superficial gray matter, deep grey matter, and the brainstem (Table S3). There was no evidence of a carry-over effect for any analyses with no association or interaction with randomization order or visit number.

Although sildenafil and cilostazol did not reduce MCA-PI, both drugs increased MCA peak systolic and end-diastolic velocities compared with placebo, with no difference between the drugs (Table 2; Figure S2). In contrast, there was a significant reduction in aortic SBP with both drugs but only sildenafil significantly reduced aortic DBP versus both placebo (−4.6 mm Hg; *P*=3.4×10^−6^) and cilostazol (−3.7 mm Hg; *P*=0.0003), although effects on cerebral blood flow velocity was not mediated by aortic effects (Table S4). As a result, both drugs reduced cerebrovascular resistance (Table 2).

Consistent with effects on TCD markers of cerebral blood flow, sildenafil increased cerebral perfusion at baseline on ASL-MRI (Figure 2; Tables S3 and S4) in white matter hyperintensities, normal-appearing white matter, gray matter, and brainstem. It did not reduce the arterial arrival time of blood. There was no significant change in cerebral perfusion or arrival time with cilostazol versus either sildenafil or placebo (Table S4).

Sildenafil was associated with an increased incidence of headaches compared with placebo, but these headaches were mostly mild (Table 3). Headache was also more common with cilostazol than placebo, and more frequent with cilostazol than sildenafil. Participants on sildenafil reported a clinically evident change in sexual function, with increased tumescence (sildenafil, 29%; cilostazol, 1.5%; placebo, 0%; *P*=1.14×10^−8^) that may have affected blinding. Cilostazol was associated with an increased incidence of diarrhea compared with placebo or sildenafil, including episodes reported as moderate to severe diarrhea. There were no serious adverse events during the study but there was 1 episode of clinically relevant bleeding on cilostazol, due to bleeding from preexisting diverticular disease. Although both drugs were well tolerated overall, there was a trend to more participants stopping medication due to adverse effects on cilostazol (*P*=0.08) with 6 (9.2%) participants stopping due to side effects on cilostazol (3 due to headache; 3 due to diarrhea), 1 (1.5%) patient on sildenafil and 2 (2.9%) participants on placebo.

**Table 3.**
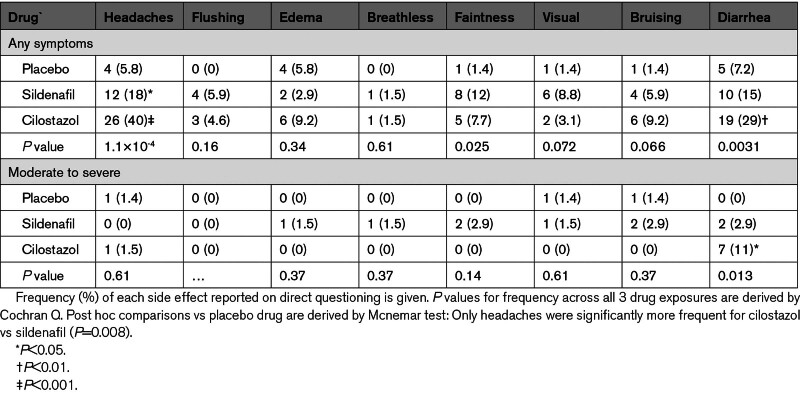
Side Effects Reported on Each Drug

## DISCUSSION

In 75 participants with mild-moderate WMH, sildenafil did not reduce pulsatility in the MCA but improved CVR on TCD, whereas cilostazol did not. However, both sildenafil, and cilostazol increased cerebral blood flow on TCD and reduced cerebrovascular resistance, with increased cerebral blood flow velocities and concurrent reductions in aortic systolic and diastolic blood pressure. The MRI substudy demonstrated consistently improved hemodynamic function with sildenafil compared with placebo with improved CVR magnitude and speed of response and an increase in cerebral perfusion. Both drugs were well tolerated despite an increased rate of mild headache, but cilostazol was associated with an increase in clinically relevant diarrhea and a nonsignificantly greater drop-out rate.

Sildenafil inhibits intracellular PDE5, prevents breakdown of cGMP and thus enhances the vascular smooth muscle response to endothelial NO release. Its hemodynamic effects in OxHARP are consistent with the expected effects of other leading candidate drugs for cSVD. ISMN targets the NO–PDE–cGMP pathway by increasing NO release, increased CVR in the small, pilot LACI-1 trial (Lacunar Intervention-1),^15^ and improved cognitive outcomes at 1 year in the LACI-2 (Lacunar Intervention-2) feasibility study, but effects on pulsatility, resistance, and absolute blood flow have not been assessed.^16^ Similarly, cilostazol targets PDE3 causing vasodilatation through increased cAMP levels in vascular smooth muscle and has pleiotropic effects on antiplatelet function and increases heart rate. It reduced the risk of recurrent stroke in cSVD in addition to standard antiplatelet therapy,^18^ improved CVR in LACI-1, reduced cerebral pulsatility after lacunar stroke in ECLIPSE trial (Effect of Cilostazol in Acute Lacunar Infarction Based on Pulsatility Index of Transcranial Doppler) and reduced functional decline in LACI-2.^17^ Furthermore, as intracerebral hemorrhage was not increased, these benefits are likely to be due to its hemodynamic rather than antiplatelet effects.^28^ Therefore, although sildenafil did not reduce cerebral pulsatility in OxHARP, it demonstrated similar hemodynamic effects to ISMN and cilostazol on CVR in LACI-1, and showed additional benefits on cerebral perfusion and cerebrovascular resistance, suggesting that it has at least a similar potential for clinical benefit.

In previous trials of PDE5 inhibitors, a single dose after lacunar stroke demonstrated a nonsignificant increase in white matter perfusion on MRI in 55 participants in Perfusion by Arterial Spin labelling following Single dose Tadalafil In Small vessel disease (PASTIS) trial.^25^ while in 20 participants in Effect of Tadalafil on Cerebral Large Arteries in Stroke (ETLAS), there was improved cerebral oxygenation but no change in TCD parameters or peripheral endothelial function.^26^ However, neither trial tested CVR and both used single drug doses. Only 1 study in Becker muscular dystrophy used longer courses of a PDE5i, and also demonstrated an improvement in cerebral perfusion on MRI.^29^ Furthermore, while cilostazol reduced cerebral pulsatility in ECLIPSE,^17^ and both cilostazol and ISMN-improved CVR in LACI-1,^15^ no trials have measured systemic aortic hemodynamics, cerebral perfusion, and CVR with TCD and MRI in the same population. As such, the mechanisms of any clinical benefit have largely been assumed.

Early studies regarded cerebral pulsatility as principally a measure of distal resistance due to a reduction in end-diastolic velocity.^30^ However, despite a weak relationship with absolute aortic pressures, cerebral pulsatility is particularly dependent on aortic pulsatility,^31^ dampened in transit to the brain.^12^ It, therefore, reflects vascular stiffening and augmentation of aortic pressures by both peripheral wave reflection^21^ and the Windkessel effect, with additional modification by the heart rate.^32^ As such, vasodilatation would be expected to reduce aortic and thus cerebral pulsatility. However, sildenafil did not reduce cerebral pulsatility in OxHARP, despite reducing cerebrovascular resistance. This reflected a balanced increase in both PSV and end-diastolic velocity, and suggests that pulsatility was principally due to aortic stiffening than distal resistance,^12^ with no significant reduction in wave reflection and augmentation of central pressures.^21^ This is in contrast to the ECLIPSE trial where 90 days of cilostazol slightly reduced MCA-PI after lacunar stroke, potentially due to adaptive changes over a longer treatment duration.^17^ However, the limited effects of vasodilatation on pulsatility implies that alternative interventions may be required to reduce cerebral pulsatility, such as increasing heart rate.^32^ In contrast, the reduction in cerebrovascular resistance and increased cerebral blood flow demonstrate that sildenafil did induce cerebral vascular smooth muscle cell relaxation, despite the blood-brain barrier. Furthermore, in reducing both DBP and increasing mean cerebral blood flow velocity, it reduced distal resistance to a greater degree than large vessel vasodilatation. This is supported by the lack of a mediating effect of sildenafil on aortic pressures in the indirect effect on mean cerebral blood flow velocity. Sildenafil also increased both the gain and speed of the cerebrovascular response to CO_2_. Although this mechanism is mediated by reducing extracellular pH independent of the endothelium, the improved CVR with sildenafil demonstrates improved efficacy of vasodilatation and potentially improved compensation for impaired endothelium-dependent stimulation of vascular smooth muscle cells (VSMCs).

A central role for hypoperfusion and impaired cerebral endothelial function in cSVD is suggested by strong associations between cSVD and reduced cerebral blood flow in the white matter,^9^ falling cerebral blood flow velocities on TCD with age, increased cerebral arterial pulsatility,^12,31,33^ impaired CVR^34^ and a moderate reduction in progression of WMH with intensive blood pressure control.^35^ Cognitive decline is also associated with hypoperfusion, postural hypotension^36^ and low diastolic blood pressure and thus diastolic flow.^37^ Therefore, the improvement in cerebral blood flow and endothelial function in cSVD with sildenafil implies it can reverse the hemodynamic dysfunction in cSVD and thus could improve clinical outcomes. Furthermore, OxHARP identified potential biomarkers of treatment effects that may be applicable in clinical practice. CO_2_-dependent CVR is technically challenging but cerebral blood flow velocity and cerebrovascular conductance index are easily measured in centers able to perform transcranial ultrasound, while perfusion MRI is available in most clinical centers.

There were limitations to this trial. First, the study was principally designed to test the superiority of sildenafil over placebo. Therefore, there were fewer MRI scans planned on cilostazol treatment and testing was commonly performed in the afternoon. As a result, observed administration of the final medication dose ensured that tests were performed at approximate peak serum concentrations of sildenafil but not peak concentrations of cilostazol as afternoon testing would occur after mid-day placebo. However, steady-state serum concentrations should have been achieved due to its longer half-life and a significant therapeutic effect would still be expected. Nonetheless, these factors may have reduced sensitivity to the effects of cilostazol. Second, treatment only lasted 3 weeks. Although this is the longest treatment duration in any randomized trial of a PDE5i in cSVD it is still unable to assess longer-term adaptive changes. Third, the population included few participants with vascular cognitive impairment and too few women for sex-specific analyses, limiting generalizability to these populations. Fourth, OxHARP did not assess effects on blood-brain barrier integrity, a key measure of endothelial dysfunction in cSVD.^38^ Fifth, despite observational associations between WMH with hypoperfusion and reduced reactivity, their improvement with sildenafil does not prove that this will result in clinical benefits in reducing the risk of stroke or cognitive decline. Any potential clinical benefit of sildenafil in improving these measures will need testing in future trials. Finally, cilostazol was more rapidly titrated than in LACI-2, which may have affected tolerability and resulted in reduced compliance. However, OxHARP has unique strengths. It was the largest physiologically guided phase 2 trial in this population; it used multimodal, extended physiological testing with TCD and MRI to define the underlying mechanisms; and it used rigorous blinding, overencapsulated placebo, and a crossover design.

Overall, the improved cerebrovascular dynamics with sildenafil provide a new potential treatment to prevent progression of cSVD that needs testing in clinical trials. Although further analysis of the tertiary outcomes in OxHARP (autonomic function, cerebral autoregulation, peripheral vascular reactivity, and interactions with blood biomarkers) may inform the mechanistic basis for the changes in cerebrovascular hemodynamics, the similar or greater physiological effects of sildenafil to cilostazol and ISMN warrants a trial to test its effects on cognitive and functional outcomes in cSVD. Furthermore, in addition to identifying new treatments, it is necessary to identify short-term biomarkers of treatment efficacy that can guide future clinical treatments. Future trials in all relevant cSVD populations should therefore ideally include both physiological testing and fluid biomarkers, with harmonized outcome measures agreed across trials.^39^

## CONCLUSIONS

Sildenafil did not reduce cerebral pulsatility compared with placebo, but it increased CVR and reduced cerebrovascular resistance, aortic blood pressure, and increased cerebral blood flow. It was noninferior to cilostazol in all comparisons, which reduced cerebrovascular resistance but did not improve CVR. Sildenafil was also better tolerated. The extensive physiological testing in OxHARP, therefore, provides a new paradigm to test the mechanisms underlying cSVD and assess potentially beneficial candidate drugs. Finally, trials of the clinical efficacy of sildenafil in cSVD are warranted.

## ARTICLE INFORMATION

### Acknowledgments

The authors are grateful first and foremost to the participants in the trial for their time and commitment to the study. The authors also gratefully thank the radiographers at the Wellcome Centre for Integrative Neuroimaging, University of Oxford and the team at the Wolfson Centre for Prevention of Stroke and Dementia, University of Oxford.

### Sources of Funding

A.J.S. Webb and the Oxford Haemodynamic Adaptation to Reduce Pulsatility trial was funded by a Wellcome Trust Clinical Research Career Development Fellowship (206589/Z/17/Z). K.A. Feakins and A.J.S. Webb were funded by an Alzheimer Society grant (450-AS-PG-18-018). A.M.K. Rothman is funded by a Wellcome Trust Clinical Research Career Development Fellowship (206632/Z17/Z). O. Llwyd is funded by a Stroke Association Postdoctoral Fellowship (21\100029). A. Lawson and C.R. Stewart were supported by the NIHR Oxford Biomedical Research Centre.

### Disclosures

A.J.S. Webb has received related funding from the Wellcome Trust, Alzheimer Society and British Heart Foundation and has received consulting fees from Woolsey Pharmaceuticals. J. Dawson is on advisory boards and has received speakers fees from AstraZeneca and Medtronic. D.J. Werring reports grant funding from the Stroke Association and British Heart Foundation; speaking honoraria from Bayer; speaking and chairing honoraria from Alexion and NovoNordisk; and consultancy fees from Alnylam, Bayer and NovoNordisk. The other authors report no conflicts.

### Supplemental Material

Trial Inclusion and Exclusion Criteria

Tables S1–S4

Figures S1–S4

## Supplementary Material


