# Cerebrovascular Effects of Sildenafil in Small Vessel Disease: The OxHARP Trial

**DOI:** 10.1161/CIRCRESAHA.124.324327

**Published:** 2024-06-04

**Authors:** Alastair JS Webb, Jacqueline Birks, Karolina A Feakins, Amy Lawson, Jesse Dawson, Alexander MK Rothman, David J Werring, Osian Llwyd, Catriona Stewart, James Thomas

**Affiliations:** 1 Wolfson Centre for Prevention of Stroke and Dementia, University of Oxford, UK; 2 Department of Brain Sciences, Imperial College London, London, UK; 3 Centre for Statistics in Medicine, Botnar Research Centre, University of Oxford, UK; 4 School of Cardiovascular and Metabolic Health, University of Glasgow, UK; 5 Department of Cardiovascular Science, University of Sheffield, UK; 6 Research Department of Brain Repair and Rehabilitation, Institute of Neurology, University College London, UK

**Keywords:** Small vessel disease, sildenafil, cilostazol, randomised controlled trial, perfusion, cerebrovascular reactivity, Vascular Disease

## Abstract

**Background:**

Vascular cognitive impairment due to cerebral small vessel disease (cSVD) is associated with cerebral pulsatility, white matter hypoperfusion and reduced cerebrovascular reactivity (CVR), and is potentially improved by endothelium-targeted drugs such as cilostazol. Whether sildenafil, a phosphodiesterase-5 inhibitor, improves cerebrovascular dysfunction is unknown.

**Methods:**

OxHARP was a double-blind, randomised, placebo-controlled, 3-way crossover trial after non-embolic cerebrovascular events with mild-moderate white matter hyperintensities (WMH), the most prevalent manifestation of cSVD. The primary outcome assessed superiority of 3 weeks of sildenafil 50mg thrice-daily versus placebo (mixed-effect linear models) on middle cerebral artery pulsatility, derived from peak systolic (PSV) and end-diastolic (EDV) velocities (transcranial ultrasound, TCD), with non-inferiority to cilostazol 100mg twice-daily. Secondary endpoints included: cerebrovascular reactivity during inhalation of air, 4% and 6% CO2 on TCD (TCD-CVR); BOLD-MRI within WMH (CVR-WMH) and normal appearing white matter (CVR-NAWM); cerebral perfusion by arterial spin labelling (MRI-pcASL); and resistance by cerebrovascular conductance. Adverse effects were compared by Cochran’s Q.

**Results:**

In 65/75 (87%) patients (median 70 years, 79% male) with valid primary outcome data, cerebral pulsatility was unchanged on sildenafil versus placebo (0.02, -0.01 to 0.05, p=0.18), or versus cilostazol (-0.01, -0.04 to 0.02, p=0.36), despite increased blood flow (ΔPSV 6.3cm/s, 3.5 to 9.07, p<0.001; ΔEDV 1.98, 0.66 to 3.29, p=0.004). Secondary outcomes improved on sildenafil versus placebo for CVR-TCD (0.83cm/s/mmHg, 0.23 to 1.42, p=0.007), CVR-WMH (0.07, 0 to 0.14, p=0.043), CVR-NAWM (0.06, 0.00 to 0.12, p=0.048), perfusion (WMH: 1.82mls/100g/min, 0.5 to 3.15, p=0.008; NAWM: 2.12, 0.66 to 3.6, p=0.006) and cerebrovascular resistance (sildenafil-placebo: 0.08, 0.05-0.10, p=4.9x10^-8^; cilostazol-placebo: 0.06, 0.03 - 0.09; p=5.1x10^-5^). Both drugs increased headaches (p=1.1x10^-4^), whilst cilostazol increased moderate-severe diarrhoea (p=0.013).

**Conclusions:**

Sildenafil did not reduce pulsatility but increased cerebrovascular reactivity and perfusion. Sildenafil merits further study to determine whether it prevents the clinical sequelae of small vessel disease.

**Trial Registration:**

Clinicaltrials.gov: NCT03855332

## Graphical abstract

**Figure:**
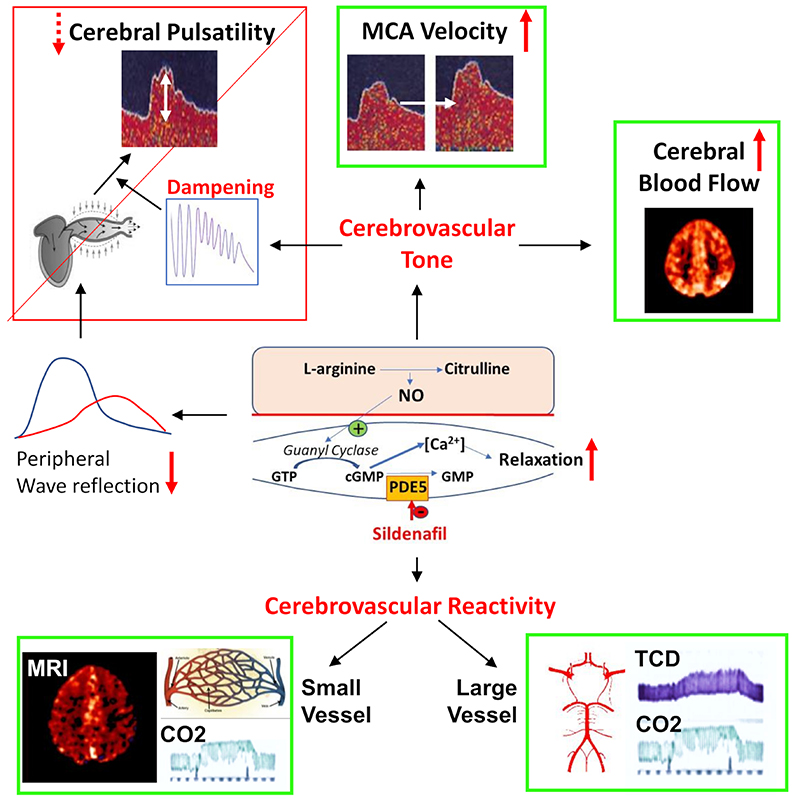
Graphical Abstract

## Introduction

Cerebral small vessel disease is due to chronic damage to the small vessels of the brain (cSVD). It is evident on MRI in over half of people over 65^1^ as dilated perivascular spaces, lacunar strokes, microbleeds or white matter hyperintensities (WMH), with WMH being the most prevalent manifestation of cSVD responsible for significant morbidity.^2^ It causes up to 30% of ischaemic strokes, 80% of haemorrhagic strokes^3^ and 40% of all-cause dementia, but there is no specific treatment^4^ due to a limited understanding of the pathophysiology of cSVD^5, 6^ and few clinical trials.^7^ cSVD is strongly associated with midlife hypertension and its long-term consequences,^8^ including reduced white matter perfusion;^9^ increased arterial stiffness;^10^ and resulting increased cerebral arterial pulsatility.^11^ Pulsatility is particularly strongly associated with cSVD,^12^ is highly reproducible and parallels disease progression.^13^ cSVD is also strongly associated with endothelial dysfunction, manifest as blood-brain barrier breakdown^11^ and reduced cerebrovascular reactivity (CVR).^5^ These modifiable physiological outcomes provide short-term targets to support translational mechanistic trials.^14^

Drugs targeting endothelium-dependent vasodilatation are leading candidates to reduce harm in cSVD. Isosorbide mononitrate (ISMN) improved cerebrovascular reactivity (CVR) in a 26 patient pilot trial,^15^ and improved cognitive outcomes in 363 patients in the LACI 2 feasibility trial.^16^ The phosphodiesterase 3 inhibitor (PDE3i) cilostazol reduced cerebral pulsatility 90 days after lacunar stroke^17^ and reduced recurrent stroke risk in cSVD patients within multiple large RCTs^18^. In contrast, the recent TREAT-SVDs trial found no difference in the physiological effects of amlodipine versus atenolol or losartan on cerebrovascular reactivity in sporadic cSVD.^19^ However, mechanistic trials in cSVD have largely assessed single physiological outcomes, limiting understanding of their physiological effects.

Phosphodiesterase 5 inhibitors (PDE5i) increase smooth muscle cGMP in response to endothelial nitric oxide release^20^ and improve endothelial dysfunction in erectile dysfunction, pulmonary arterial hypertension and Raynaud’s Disease. By enhancing the smooth muscle response to endothelial nitric oxide they are predicted to improve blood flow and increase CVR, whilst vasodilatation could reduce blood pressure augmentation,^21, 22^ improve dampening of the arterial waveform and thus reduce pulsatility. They have been associated with reduced dementia in large populations^23^ and amyloid-independent benefits in animal models,^24^ but there are no mechanistic studies of their sustained haemodynamic effect in cSVD.^25,26^ However, given the widespread use of PDE5i, their excellent tolerability, minimal interactions with established preventative treatments and a biologically plausible mechanism, they are good candidates to reduce the clinical sequelae of cSVD.

The OxHARP randomised, double-blind, placebo-controlled crossover clinical trial established a physiological paradigm to test the effects of sildenafil on cerebral arterial pulsatility and cerebrovascular reactivity in patients with symptomatic mild to moderate small vessel disease, testing superiority compared to placebo and non-inferiority to cilostazol.

## Methods

### Study Design

OxHARP was a double-blind, randomised, placebo-controlled, 3-way crossover phase 2 trial with physiological endpoints^27^, that ran from 11th July 2019 to the last visit of the last patient on 6th December 2022, with a 6 month pause due to the COVID-19 pandemic from March to September 2020. All procedures were performed at the Wolfson Centre for Prevention of Stroke and Dementia. OxHARP is sponsored by the University of Oxford, approved by the UK Health Research Authority and South Central – Oxford C Research Ethics Committee (19/SC/0022), and is registered with ClinicalTrials.org (NCT03855332). The study protocol has been published previously.^27^

### Participants

OxHARP aimed to include 75 participants with a previous cryptogenic or lacunar stroke or TIA requiring secondary preventative treatment, with mild to moderate WMH evident on their most recent clinical brain imaging within the past 6 years (Fazekas score on MRI or modified Blennow score on CT of 1-3 below 60 or 1-4 above 60), allowing for 15% drop-out. Inclusion and exclusion criteria are provided in supplemental data. Following referral from stroke services or identification via a research registry, potential participants were screened, provided face-to-face written consent, and gave demographic, clinical and cognitive data. Transcranial ultrasound was performed at screening to confirm adequate temporal bone windows for measurement of cerebral pulsatility index. The full inclusion criteria have been reported previously.^27^ All participants without a contraindication to MRI were consecutively invited to join the MRI substudy. This initially aimed to recruit 30 participants to be scanned on sildenafil and placebo but the trial was amended to recruit up to a further 30 participants to undergo MRI on all three treatments in May 2020. Participants who did not tolerate MRI were eligible to continue with TCD alone.

### Randomisation

The sequence of drug treatments was randomly allocated pre-treatment by study number by the manufacturing pharmacy, stratified by MRI substudy. Medications were dispensed by the clinical trial pharmacy independently of the blinded study team, in scheduled treatment packs containing over-encapsulated, identical medications. All participants, researchers and study physicians were blinded to drug allocation. To assess unblinding, men were asked about change in sexual activity and tumescence. The drug allocation code was held by the dispensing trial pharmacy in case of serious adverse events requiring medical intervention.

### Procedures

Potential participants underwent a telephone screening followed by a face-to-face screening visit at least 1 week later. The baseline assessment was completed either at screening or within 1 month. A standardised demographic, clinical and medication history and clinical examination was performed by a study physician, including baseline cognitive testing (Montreal Cognitive Assessment, digit-symbol coding task, Fluid Intelligence task), blood tests and ECG. At baseline and all follow-up visits, a standardized physiological assessment was performed. MRI was performed at follow-up visits in the MRI sub-study.

Following each visit, participants received treatment in randomized order with overencapsulated, double blind medication. Each treatment lasted for three weeks, starting with either thrice-daily placebo, thrice-daily sildenafil 25mg, or twice daily cilostazol 50mg with a placebo dose at midday. After one week, the dose was doubled by taking two tablets at each dose, unless limited by side effects. There was a minimum 1 week wash out between drugs. Assessments were performed on the final day of treatment.

Physiological assessments were performed in a temperature controlled laboratory (21-23°C) after 20 minutes supine rest, timed to be 30 minutes after observed administration of trial medication in clinic. Middle cerebral artery flow velocity was assessed by transcranial ultrasound via the transtemporal window to derive the primary outcome: Gosling’s Pulsatility Index (peak systolic velocity – end-diastolic velocity) / mean flow velocity). The principal secondary outcome was cerebrovascular reactivity (CVR), assessed during bilateral TCD monitoring of the middle cerebral artery with concurrent ECG, non-invasive blood pressure monitoring calibrated to an oscillometric brachial reading (FMS, Finometer Midi) and end-tidal carbon dioxide monitoring (etCO2, AD Instruments Gas Analyser ML206). After 10 minutes rest, CVR was assessed during 2-minute alternating periods of inhalation of medical air, 4% CO2 and 6% CO2, delivered via a respiratory circuit with a well-sealed, non-invasive ventilation mask. Aortic blood pressure was determined by radial artery applanation tonometry (Sphygmocor).^27^

After completion of physiological testing, participants in the MRI substudy underwent structural MRI including T1, FLAIR, susceptibility weighted imaging, and diffusion tensor imaging, distributed between the first two MRI visits. CVR on MRI was assessed at each MRI follow-up on multi-band Blood oxygen level dependent (BOLD) MRI with whole brain acquisition every 800ms, with perfusion imaging with pseudo-continuous Arterial Spin Labelling (pcASL). Details of the imaging protocol have been reported previously.^27^ During CVR, participants breathed medical air or 6% CO2 (balance medical air) in 2 sets of 2 minute alternating periods, delivered by the same respiratory circuit used during CVR assessment with TCD. Throughout imaging, participants had continuous non-invasive monitoring of etCO2 (AD Instruments Gas Analyser ML206), respiratory motion, oxygen saturations and continuous blood pressure.

### Outcomes

The primary outcome was Gosling’s middle cerebral artery pulsatility (MCA-PI, (PSV-EDV) / mean flow velocity)) on transcranial ultrasound, derived from the average of 3 manually measured peak systolic and 3 end-diastolic velocities, from each of two recordings, reviewed and quality assessed by 2 blinded reviewers (AJSW, JT). Where both MCA-PI recordings were of similar quality, with no measurement artefacts and a similar mean velocity (within 10%), an average was taken. Otherwise, the higher quality recording was used. Disagreements between reviewers were resolved by a panel discussion. The secondary outcome was CVR on TCD, with the optimal side selected by the same process as for MCA-PI, with 2 blinded reviewers (AJSW, OL). This was defined as change in mean flow velocity per mmHg change in etCO2 from the beta-coefficient from a linear model between the etCO2 value during inhalation of medical air, 4% and 6% CO2 and mean MCA flow velocity, after correction for phase delay by cross-correlation with piecewise cubic Hermitte interpolation. Tertiary outcomes included effects of each drug on aortic blood pressure, on the physiological measurements used to derive the primary and secondary outcomes (PSV, EDV, MFV) and on the relationship between them as a measure of cerebrovascular resistance, estimated as the cerebrovascular conductance index (CVCi= MFV / aortic MBP).

BOLD-CVR images were pre-processed (MCFLIRT, B0 unwarping (BBR), high-pass temporally filtered at 300s and brain extracted). Regions of interest were defined conservatively by segmentation of T1 images by tissue type (fMRIB Software Library, FSL: FAST/FIRST), erosion by 1 voxel and segmentation into white matter hyperintensities (WMH) and normal appearing white matter (NAWM, FSL:BIANCA), followed by registration to structural and standard space (FLIRT and FNIRT). Phase delays between recorded etCO2 and each voxel timeseries were estimated by cross-correlation to the maximum r^2^ (Matlab, in-house software) and phase-shifted by piecewise cubic Hermitte interpolation. Associations between phase-shifted etCO2 timeseries and each voxel are determined by general linear models on a voxel-wise basis (matlab) and by FEAT (FSL), expressing CVR as the average percentage change in BOLD response per mmHg change in etCO2 across all voxels in the regions of interest (NAWM and WMH). In a sensitivity analysis, the average voxel-wise, within-individual difference for each MRI outcome is determined following registration to standard space. WMH volume on MRI was quantified for OxHARP trial scans from FLAIR images (BIANCA, FSL).

Safety and adverse events were assessed face-to-face by a study physician at each visit, including standardized assessment of the most frequent adverse events (headache, flushing, oedema, breathlessness, light-headedness, visual disturbance, bruising/bleeding, diarrhea, priapism). All adverse events were reviewed by the study chief investigator (AW) for assignment of severity and causality, before unblinding. SAEs were reported to the independent DSMB within 24 hours, and the DSMB met every 6 months for blinded review of recruitment and adverse events rates, with unblinded data review if requested.

### Statistical Analysis

The primary drug comparisons used mixed effect linear models, adjusted for age, sex, visit order and allocation randomisation sequence, with the primary study outcome defined as the difference between sildenafil versus placebo on cerebral pulsatility (MCA-PI). Secondary outcomes included CVR on TCD and CVR in WMH and NAWM on MRI. Non-inferiority of sildenafil versus cilostazol on MCA-PI was assessed by the lower margin of the confidence interval compared to the pre-defined non-inferiority threshold (0.08 for MCA-PI). Cilostazol was compared to placebo as for the primary outcome. The primary analysis used mixed effect linear models to allow for missing data due to medication side effects and increased time intervals due to COVID. Mediation analysis was used to assess whether drug effects on cerebral transcranial ultrasound indices were mediated by effects on aortic blood pressure, and whether cerebrovascular reactivity on MRI was mediated by effects on the MCA. Rates of adverse events were compared across all treatments in participants receiving all three treatments by Cochran’s Q, with post-hoc pairwise comparisons by McNemar’s test.

All analyses were performed in R, matlab or stata. The protocol and statistical analysis plan have been published previously.

The funder of the study had no role in study design, data collection, data analysis, data interpretation, or writing of the report.

## Results

**Figure 1:**
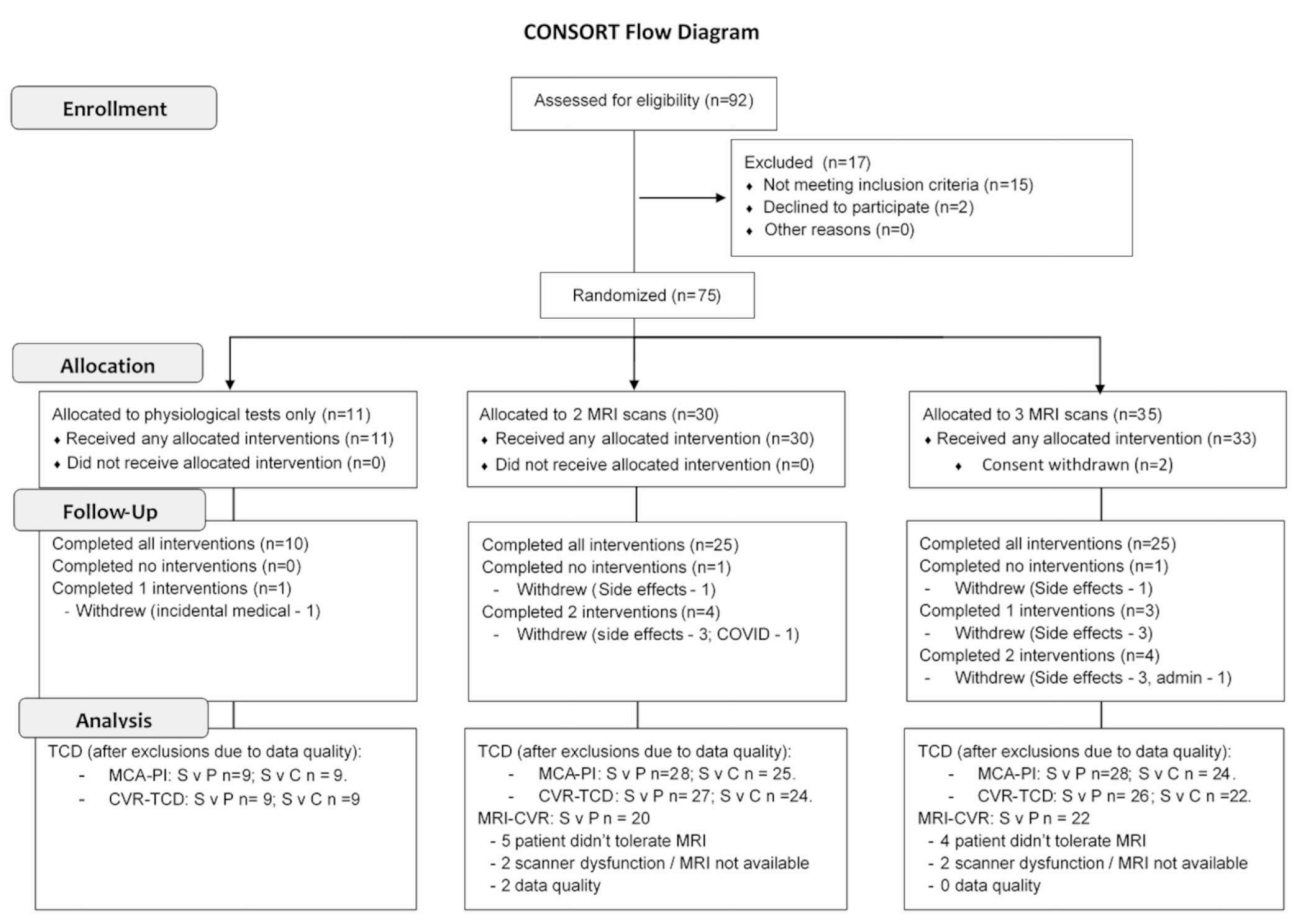
Consort Flow Diagram

**Figure 2:**
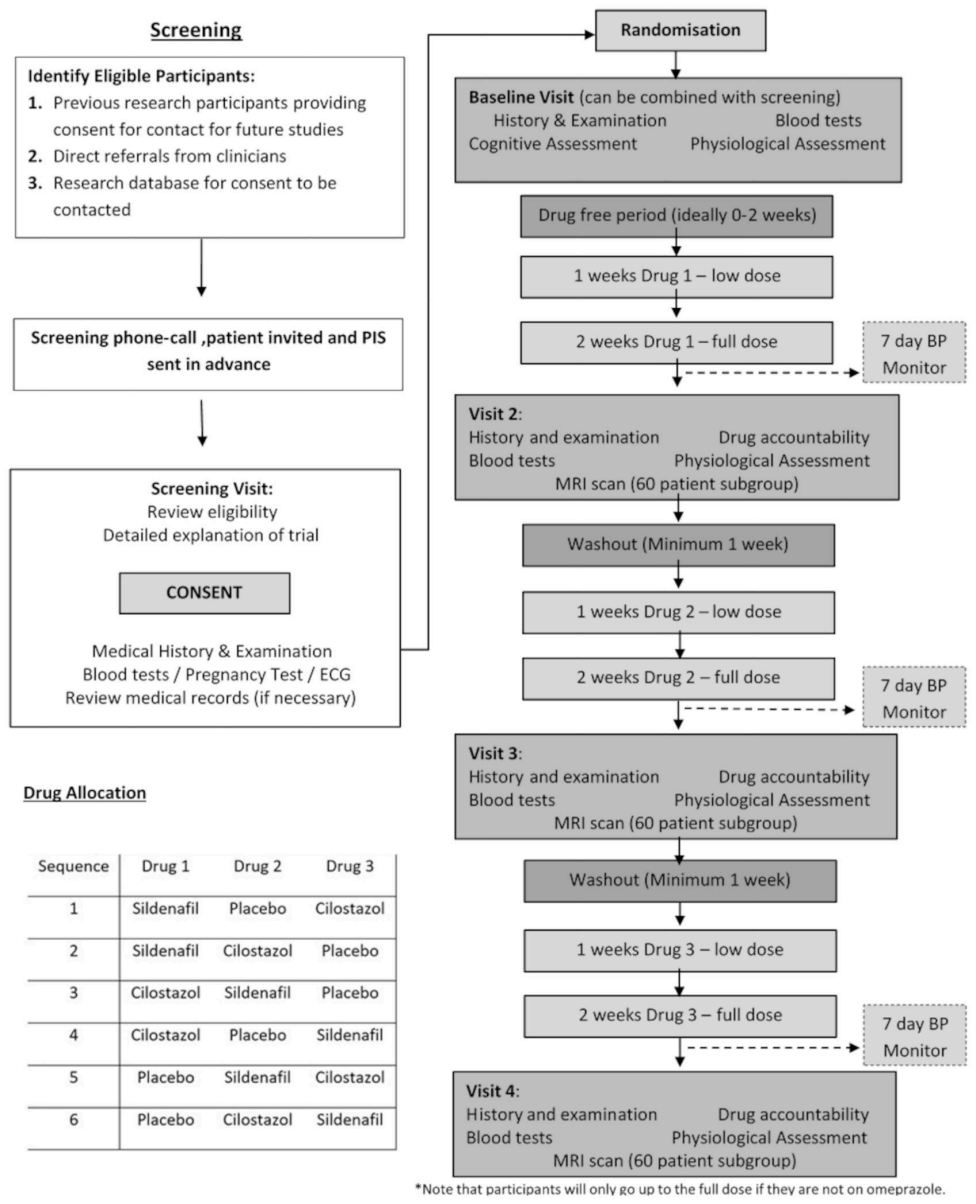
Study Flow Diagram

Of 92 potential participants screened face-to-face, 17 participants were not eligible due to comorbidities (figure 1), whilst 2 randomised participants withdrew before receiving any medication. 35 participants consented to 3 MRIs, 30 to 2 MRIs and 10 participants only to TCD assessment. 65 (89%) participants had primary outcome data, 63 (86%) had valid data for the CVR comparison by TCD and 43 (59%) had valid data for sildenafil versus placebo on MRI (figures 1-2). The OxHARP population had a median age of 70, the majority were men (78%) and had a previous stroke (60%), with nearly equal participants with mild (53%) versus moderate (26%) or moderately-severe (20%) white matter hyperintensities (table 1). Participants opting only to undergo physiological testing were slightly older, with lower CVR and cerebral blood flow velocities.

**Figure 3:**
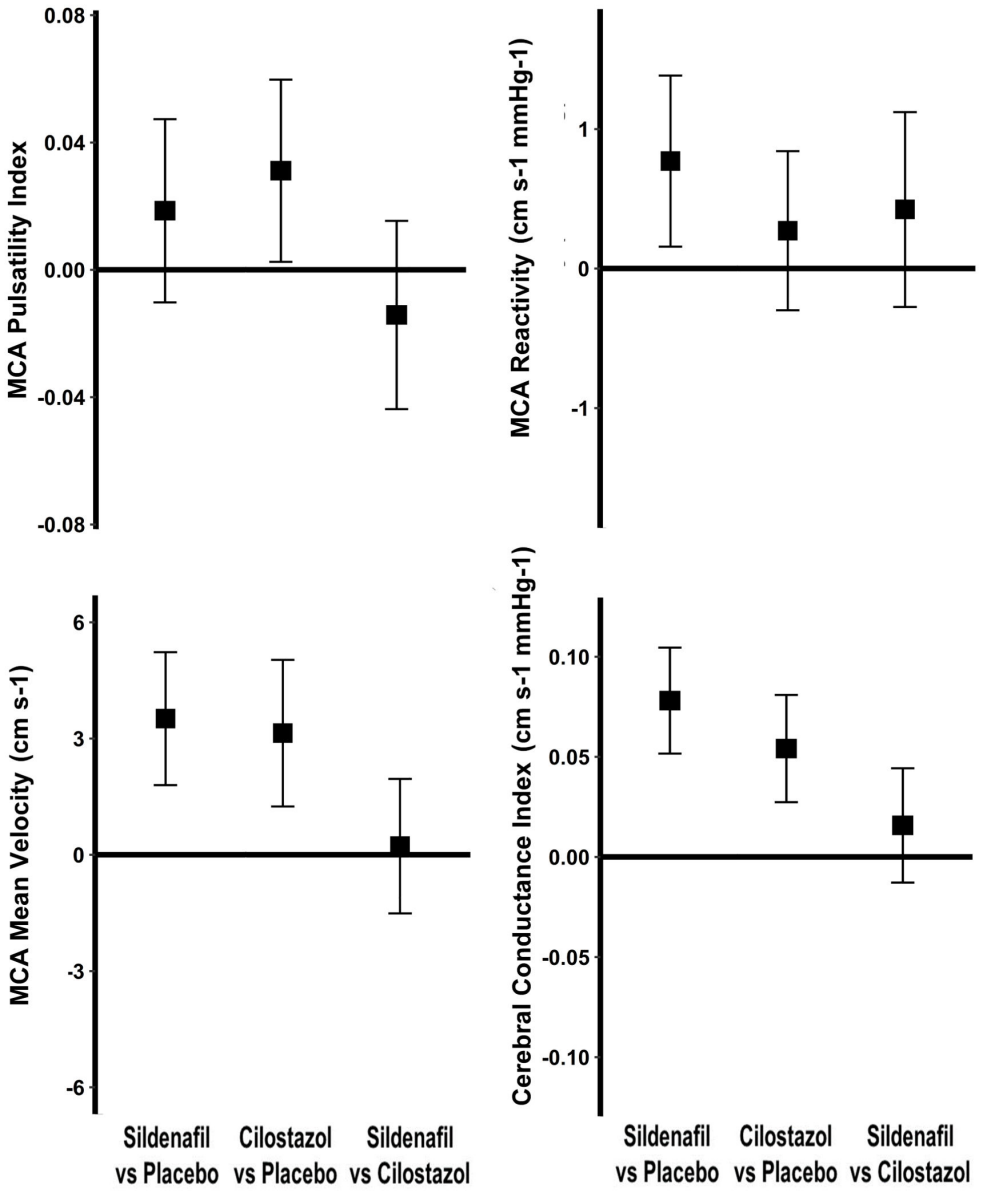
Differences between sildenafil, cilostazol and placebo on middle cerebral artery blood flow, pulsatility and reactivity on transcranial ultrasound. The mean, within-individual difference for the first drug versus the second is shown with 95% confidence intervals for: A) middle cerebral artery pulsatility (MCA-PI); B) cerebrovascular reactivity from the beta-coefficient from a linear model for the association between end-tidal CO2 and mean flow velocity (CVR); C) Mean MCA flow velocity (MFV); D) Cerebrovascular conductance index (CVCi - MFV / aortic MBP). Differences are shown for sildenafil minus placebo (S-P), sildenafil minus cilostazol (S-C) and cilostazol minus placebo (C-P).

There was no significant difference between the effect of sildenafil and placebo on MCA-PI (table 2, figure 3), including when analysed as difference from baseline. Sildenafil was non-inferior to cilostazol (upper confidence interval = 0.02 compared to the non-inferiority threshold of 0.08), but there was a significant increase in MCA-PI on cilostazol versus placebo (table 2, figure 3). There was no evidence of a carry over effect with no association or interaction with randomisation order or visit number.

**Figure 4:**
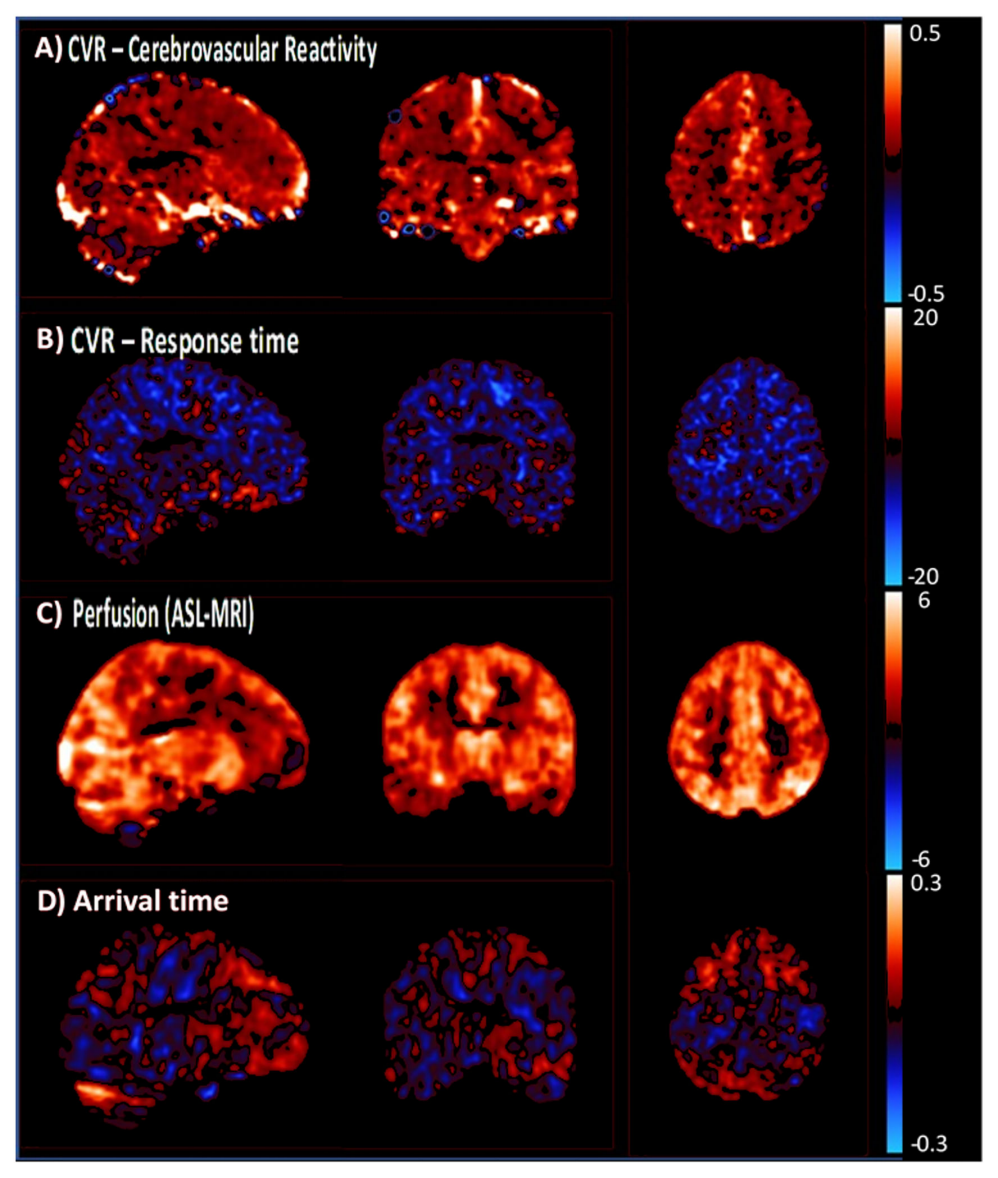
Differences between sildenafil and placebo on cerebrovascular reactivity and cerebral blood flow on MRI. The mean, within-individual difference on MRI imaging for sildenafil minus placebo is shown on a voxel-wise basis, following registration to standard MNI space and Gaussian smoothing. Results are shown for A) CVR: cerebrovascular reactivity as percentage change in BOLD per mmHg end-tidal CO2); B) CVR response: difference in time delay between etCO2 and BOLD signal in seconds; C) Difference in perfusion on ASL, in mls/min/100g; D) Difference in arrival time of blood flow during ASL, in seconds.

Sildenafil significantly increased TCD-CVR in the middle cerebral artery compared to placebo (p=0.0071, table 2, figure 3). This was consistent with increased CVR on sildenafil versus placebo on MRI within white matter hyperintensities and normal appearing white matter, after adjustment for confounders (figure 4, supplemental tables 3-4), although with only a trend to increased CVR in unadjusted comparisons (supplemental table 3). Effects on parenchymal reactivity on MRI may have been partially mediated by effects on the middle cerebral artery, but with only borderline significance (supplemental table 4). There was no significant difference between sildenafil and cilostazol on TCD-CVR (+0.47, p=0.14). In addition to the increased magnitude of CVR with sildenafil, there was a more rapid CVR response to carbon dioxide with sildenafil versus placebo (figure 2, supplemental table 4). There was no difference between cilostazol with either placebo or sildenafil on MRI (supplemental table 3-4, supplemental figures 4-5), but the study was underpowered for the comparison of cilostazol and placebo on MRI, with only 15 participants having MRI scans on both. In other tissues, CVR was greater with sildenafil than placebo in superficial grey matter, deep grey matter and the brainstem (supplemental table 3). There was no evidence of a carry over effect for any analyses with no association or interaction with randomisation order or visit number.

Although sildenafil and cilostazol did not reduce MCA-PI, both drugs increased middle cerebral artery peak systolic and end-diastolic velocities compared to placebo, with no difference between the drugs (table 2, supplemental figure 2). In contrast, there was a significant reduction in aortic SBP with both drugs but only sildenafil significantly reduced aortic DBP versus both placebo (-4.6mmHg, p=3.4x10^-6^) and cilostazol (-3.7mmHg, p=0.0003), although effects on cerebral blood flow velocity was not mediated by aortic effects (supplemental table 4). As a result, both drugs reduced cerebrovascular resistance (table 2).

Consistent with effects on TCD markers of cerebral blood flow, sildenafil increased cerebral perfusion at baseline on ASL-MRI (figure 2, supplemental table 3-4) in white matter hyperintensities, normal-appearing white matter, grey matter and brainstem. It did not reduce the arterial arrival time of blood. There was no significant change in cerebral perfusion or arrival time with cilostazol versus either sildenafil or placebo (supplemental table 4).

Sildenafil was associated with an increased incidence of headaches compared to placebo, but these headaches were mostly mild (table 3). Headache was also more common with cilostazol than placebo, and more frequent with cilostazol than sildenafil. Participants on sildenafil reported a clinically evident change in sexual function, with increased tumescence (sildenafil 29%; cilostazol 1.5%; placebo 0%; p=1.14x10^-8^) that may have affected blinding. Cilostazol was associated with an increased incidence of diarrhoea compared to placebo or sildenafil, including episodes reported as moderate to severe diarrhoea. There were no serious adverse events during the study but there was one episode of clinically relevant bleeding on cilostazol, due to bleeding from pre-existing diverticular disease. Although both drugs were well tolerated overall, there was a trend to more participants stopping medication due to adverse effects on cilostazol (p=0.08) with 6 (9.2%) participants stopping due to side effects on cilostazol (3 due to headache; 3 due to diarrhoea), 1 (1.5%) patient on sildenafil and 2 (2.9%) participants on placebo.

## Discussion

In 75 participants with mild-moderate WMH, sildenafil did not reduce pulsatility in the middle cerebral artery but improved cerebrovascular reactivity on TCD, whereas cilostazol did not. However, both sildenafil and cilostazol increased cerebral blood flow on TCD and reduced cerebrovascular resistance, with increased cerebral blood flow velocities and concurrent reductions in aortic systolic and diastolic blood pressure. The MRI substudy demonstrated consistently improved haemodynamic function with sildenafil compared to placebo with improved CVR magnitude and speed of response and an increase in cerebral perfusion. Both drugs were well tolerated despite an increased rate of mild headache, but cilostazol was associated with an increase in clinically relevant diarrhoea and a non-significantly greater drop out rate.

Sildenafil inhibits intracellular PDE5, prevents breakdown of cGMP and thus enhances the vascular smooth muscle response to endothelial nitric oxide release. It’s haemodynamic effects in OxHARP are consistent with the expected effects of other leading candidate drugs for cSVD. ISMN targets the nitric oxide–PDE–cGMP pathway by increasing NO release, increased cerebrovascular reactivity in the small, pilot LACI-1 trial,^15^ and improved cognitive outcomes at one year in the LACI-2 feasibiliy study, but effects on pulsatility, resistance and absolute blood flow have not been assessed.^16^ Similarly, cilostazol targets PDE3 causing vasodilatation through increased cAMP levels in vascular smooth muscle and has pleiotropic effects on antiplatelet function and increases heart rate. It reduced the risk of recurrent stroke in cSVD in addition to standard antiplatelet therapy,^18^ improved CVR in LACI-1, reduced cerebral pulsatility after lacunar stroke in ECLIPSE and reduced functional decline in LACI-2.^17^ Furthermore, as intracerebral haemorrhage was not increased, these benefits are likely to be due to its haemodynamic rather than antiplatelet effects.^28^ Therefore, although sildenafil did not reduce cerebral pulsatility in OxHARP, it demonstrated similar haemodynamic effects to ISMN and cilostazol on CVR in LACI-1, and showed additional benefits on cerebral perfusion and cerebrovascular resistance, suggesting that it has at least a similar potential for clinical benefit.

In previous trials of PDE5 inhibitors, a single dose after lacunar stroke demonstrated a non-significant increase in white matter perfusion on MRI in 55 participants in PASTIS,^25^ whilst in 20 participants in ETLAS-1 there was improved cerebral oxygenation but no change in TCD parameters or peripheral endothelial function.^26^ However, neither trial tested CVR and both used single drug doses. Only one study in Becker muscular dystrophy used longer courses of a PDE5i, and also demonstrated an improvement in cerebral perfusion on MRI.^29^ Furthermore, whilst cilostazol reduced cerebral pulsatility in ECLIPSE,^17^ and both cilostazol and ISMN improved CVR in LACI-1,^15^ no trials have measured systemic aortic haemodynamics, cerebral perfusion and cerebrovascular reactivity with TCD and MRI in the same population. As such, the mechanisms of any clinical benefit have largely been assumed.

Early studies regarded cerebral pulsatility as principally a measure of distal resistance due to a reduction in EDV.^30^ However, despite a weak relationship with absolute aortic pressures, cerebral pulsatility is particularly dependent on aortic pulsatility,^31^ dampened in transit to the brain.^12^ It therefore reflects vascular stiffening and augmentation of aortic pressures by both peripheral wave reflection^21^ and the Windkessel effect, with additional modification by the heart rate.^32^ As such, vasodilatation would be expected to reduce aortic and thus cerebral pulsatility. However, sildenafil did not reduce cerebral pulsatility in OxHARP, despite reducing cerebrovascular resistance. This reflected a balanced increase in both PSV and EDV, and suggests that pulsatility was principally due to aortic stiffening than distal resistance,^12^ with no significant reduction in wave reflection and augmentation of central pressures.^21^ This is in contrast to the ECLIPSE trial where 90 days of cilostazol slightly reduced MCA-PI after lacunar stroke, potentially due to adaptive changes over a longer treatment duration.^17^ However, the limited effects of vasodilatation on pulsatility implies that alternative interventions may be required to reduce cerebral pulsatility, such as increasing heart rate.^32^ In contrast, the reduction in cerebrovascular resistance and increased cerebral blood flow demonstrate that sildenafil did induce cerebral vascular smooth muscle cell relaxation, despite the blood brain barrier. Furthermore, in reducing both DBP and increasing MFV, it reduced distal resistance to a greater degree than large vessel vasodilatation. This is supported by the lack of a mediating effect of sildenafil on aortic pressures in the indirect effect on MFV. Sildenafil also increased both the gain and speed of the cerebrovascular response to CO2. Although this mechanism is mediated by reducing extracellular pH independent of the endothelium, the improved CVR with sildenafil demonstrates improved efficacy of vasodilatation and potentially improved compensation for impaired endothelium-dependent stimulation of VSMCs.

A central role for hypoperfusion and impaired cerebral endothelial function in cSVD is suggested by strong associations between cSVD and reduced cerebral blood flow in the white matter,^9^ falling cerebral blood flow velocities on TCD with age, increased cerebral arterial pulsatility,^12, 33, 34^ impaired cerebrovascular reactivity^35^ and a moderate reduction in progression of WMH with intensive blood pressure control.^36^ Cognitive decline is also associated with hypoperfusion, postural hypotension^37^ and low diastolic blood pressure and thus diastolic flow.^38^ Therefore, the improvement in cerebral blood flow and endothelial function in cSVD with sildenafil implies it can reverse the haemodynamic dysfunction in cSVD and thus could improve clinical outcomes. Furthermore, OxHARP identified potential biomarkers of treatment effects that may be applicable in clinical practice. CO2 dependent CVR is technically challenging but cerebral blood flow velocity and CVCi are easily measured in centres able to perform transcranial ultrasound, whilst perfusion MRI is available in most clinical centres.

There were limitations to this trial. The study was principally designed to test the superiority of sildenafil over placebo. Therefore, there were fewer MRI scans planned on cilostazol treatment and testing was commonly performed in the afternoon. As a result, observed administration of the final medication dose ensured that tests were performed at approximately peak serum concentrations of sildenafil but not peak concentrations of cilostazol as afternoon testing would occur after mid-day plaebo. However, steady state serum concentrations should have been achieved due to its longer half-life and a significant therapeutic effect would still be expected. Nonetheless, these factors may have reduced sensitivity to the effects of cilostazol. Secondly, treatment only lasted 3 weeks. Although this is the longest treatment duration in any randomised trial of a PDE5i in cSVD it is still unable to assess longer-term adaptive changes. Thirdly, the population included few participants with vascular cognitive impairment and too few women for sex-specific analyses, limiting generalisability to these populations. Fourthly, OxHARP did not assess effects on blood-brain barrier integrity, a key measure of endothelial dysfunction in cSVD.^39^ Fifthly, despite observational associations between WMH with hypoperfusion and reduced reactivity, their improvement with sildenafil does not prove that this will result in clinical benefits in reducing the risk of stroke or cognitive decline. Any potential clinical benefit of sildenafil in improving these measures will need testing in future trials. Finally, cilostazol was more rapidly titrated than in LACI-2, which may have affected tolerability and resulted in reduced compliance. However, OxHARP has unique strengths. It was the largest physiologically guided phase 2 trial in this population; it used multimodal, extended physiological testing with TCD and MRI to define the underlying mechanisms; and it used rigorous blinding, overencapsulated placebo and a crossover design.

Overall, the improved cerebrovascular dynamics with sildenafil provides a new potential treatment to prevent progression of cSVD that needs testing in clinical trials. Although further analysis of the tertiary outcomes in OxHARP (autonomic function, cerebral autoregulation, peripheral vascular reactivity, and interactions with blood biomarkers) may inform the mechanistic basis for the changes in cerebrovascular haemodynamics, the similar or greater physiological effects of sildenafil to cilostazol and ISMN warrants a trial to test its effects on cognitive and functional outcomes in cSVD. Furthermore, in addition to identifying new treatments, it is necessary to identify short term biomarkers of treatment efficacy that can guide future clinical treatments. Future trials in all relevant cSVD populations should therefore ideally include both physiological testing and fluid biomarkers, with harmonised outcome measures agreed across trials.^40^

## Conclusions

Sildenafil did not reduce cerebral pulsatility compared to placebo, but it increased cerebrovascular reactivity and reduced cerebrovascular resistance, aortic blood pressure and increased cerebral blood flow. It was non-inferior to cilostazol in all comparisons, which reduced cerebrovascular resistance but did not improve cerebrovascular reactivity. Sildenafil was also better tolerated. The extensive physiological testing in OxHARP therefore provides a new paradigm to test the mechanisms underlying cSVD and assess potentially beneficial candidate drugs. Finally, trials of the clinical efficacy of sildenafil in cSVD are warranted.

## Supplementary Material

324327 Data Supplement

324327 Graphical Abstract

324327 Major Resources Table

## Figures and Tables

**Table 1:** Clinical characteristics of population included in the OxHARP trial. TIA=transient ischaemic attack; WMH = white matter hyperintensities; MoCA = Montreal Cognitive Assessment; BP = blood pressure; CVR-TCD = cerebrovascular reactivity on transcranial ultrasound; PSV = peak systolic velocity; EDV = end diastolic velocity.

Variables	All participants
N	75
Age	70 (7.7)
Male	59 (78)
Diabetes	5 (6)
Hypertension	57 (76)
Smoker	8 (10)
White	74 (98)
Alcohol (units)	10 (1 - 20)
**Event**	
TIA	30 (40)
Stroke	45 (60)
**WMH severity**	
Mild	40 (53)
Moderate	20 (26)
Moderate-Severe	15 (20)
Systolic BP (mmHg)	127 (18)
Diastolic BP (mmHg)	70 (10)
MoCA	27 (25 - 28)
Pulsatility Index	1.00 (0.16)
CVR-TCD (cm/s/mmHg)	6.9 (2.3)
PSV (cm/s)	79.1 (17)
EDV (cm/s)	32.1 (8.4)

**Table 2:** Differences between sildenafil, placebo and cilostazol on cerebral pulsatility, cerebrovascular reactivity and associated physiological measures. Mean differences for the first agent minus the second agent are shown, with 95% confidence intervals and p-values determined by mixed effects linear models, adjusted for age, gender, visit and randomisation order. Results are shown for: middle cerebral artery pulsatility (MCA-PI), peak (PSV), trough (EDV) and mean cerebral blood flow velocity (MFV); cerebrovascular conductance index (MFV / aortic mean BP; CVCi)); and cerebrovascular reactivity (CVR) as linear change in MFV per change in end-tidal CO2 on transcranial ultrasound (TCD; m/s/mmHg), and on BOLD MRI (%/mmHg) in white matter hyperintensities (WMH) and normal appearing white matter (NAWM).

	Sildenafil vs Placebo	p-val	Cilostazol vs Placebo	p-val	Sildenafil vs Cilostazol	p-val
N	65		60		59	
**MCA blood flow**						
MCA – PI	0.02 (-0.01, 0.05)	0.18	0.03 (0, 0.06)	0.028	-0.01 (-0.04, 0.02)	0.36
PSV (m/s)	6.30 (3.53, 9.07)	1.8x10^-5^	6.42 (3.53, 9.31)	2.8x10^-5^	-0.12 (-3.00, 2.76)	0.93
EDV (m/s)	1.98 (0.66, 3.29)	0.0038	1.53 (0.16, 2.9)	0.031	0.45 (-0.92, 1.81)	0.52
MFV (m/s)	3.41 (1.72, 5.11)	0.00013	3.18 (1.41, 4.95)	0.00061	0.23 (-1.53, 1.99)	0.8
CVCi (m/s/mmHg)	0.08 (0.05, 0.1)	4.9x10^-8^	0.06 (0.03, 0.09)	5.1x10^-5^	0.02 (-0.01, 0.05)	0.17
Aortic SBP (mmHg)	-7.8 (-11.06, -4.55)	7.0x10^-6^	-7.47 (-10.86, -4.08)	3.3x10^-5^	-0.33 (-3.71, 3.05)	0.85
Aortic DBP (mmHg)	-4.6 (-6.45, -2.75)	3.4x10^-6^	-0.9 (-2.83, 1.03)	0.36	-3.7 (-5.63, -1.78)	0.0003

**Cerebrovascular Reactivity**						
CVR – TCD (m/s/mmHg)	0.83 (0.23, 1.42)	0.0071	0.41 (-0.21, 1.02)	0.20	0.42 (-0.2, 1.04)	0.18
CVR – WMH (%/mmHg)	0.07 (0.00, 0.14)	0.043	0.06 (-0.15, 0.28)	0.56	0.01 (-0.19, 0.22)	0.91
CVR – NAWM (%/mmHg)	0.06 (0.00, 0.12)	0.048	0.04 (-0.10, 0.18)	0.54	0.03 (-0.14, 0.2)	0.74
						

**Table 3:** Side effects reported on each drug. Frequency (%) of each side-effect reported on direct questioning are given. p-values for frequency across all three drug exposures are derived by Cochran’s Q. Post-hoc comparisons versus placebo drug are derived by Mcnemar’s test: * p<0.05; ** p<0.01; *** p<0.001. Only headaches were significantly more frequent for cilostazol versus sildenafil (p=0.008).

Drug`	Headaches	Flushing	Oedema	Breathless	Faintness	Visual	Bruising	Diarrhoea
**Any Symptoms**								
**Placebo**	4 (5.8)	0 (0)	4 (5.8)	0 (0)	1 (1.4)	1 (1.4)	1 (1.4)	5 (7.2)
**Sildenafil**	12 (18)*	4 (5.9)	2 (2.9)	1 (1.5)	8 (12)	6 (8.8)	4 (5.9)	10 (15)
**Cilostazol**	26 (40)***	3 (4.6)	6 (9.2)	1 (1.5)	5 (7.7)	2 (3.1)	6 (9.2)	19 (29)**
**p-value**	1.1x10^-4^	0.16	0.34	0.61	0.025	0.072	0.066	0.0031
**Moderate - Severe**								
**Placebo**	1 (1.4)	0 (0)	0 (0)	0 (0)	0 (0)	1 (1.4)	1 (1.4)	0 (0)
**Sildenafil**	0 (0)	0 (0)	1 (1.5)	1 (1.5)	2 (2.9)	1 (1.5)	2 (2.9)	2 (2.9)
**Cilostazol**	1 (1.5)	0 (0)	0 (0)	0 (0)	0 (0)	0 (0)	0 (0)	7 (11)*
**p-value**	0.61	-	0.37	0.37	0.14	0.61	0.37	0.013

## Data Availability

The data that support the findings of this study are available from the corresponding author upon reasonable request.. Proposals should be directed to alastair.webb@ndcn.ox.ac.uk; to gain access, data requestors will need to sign a data access agreement.

## Novelty and Significance

### What is known?

- Cerebral small vessel disease is associated with 30% of ischemic stroke, 80% of hemorrhagic stroke and approximately 40% of dementia, but has no specific treatment.
- Small vessel disease is associated with cerebral pulsatility, white matter hypoperfusion and reduced cerebrovascular reactivity, and these mechanisms, and associated clinical outcomes, may be improved by drugs targeting the endothelium, such as isosorbide mononitrate and cilostazol.
- Sildenafil, a phosphodiesterase 5 inhibitor, is an effective systemic vasodilator that targets endothelial function, but its cerebrovascular effects and potential in small vessel disease is unknown.
