# Supplementary figures and images for: Cerebrovascular Effects of Sildenafil in Small Vessel Disease: The OxHARP Trial
Source: Circ Res. Author manuscript; Available in PMC 2024 Jul 9. (PMC11227301; doi:10.1161/CIRCRESAHA.124.324327)

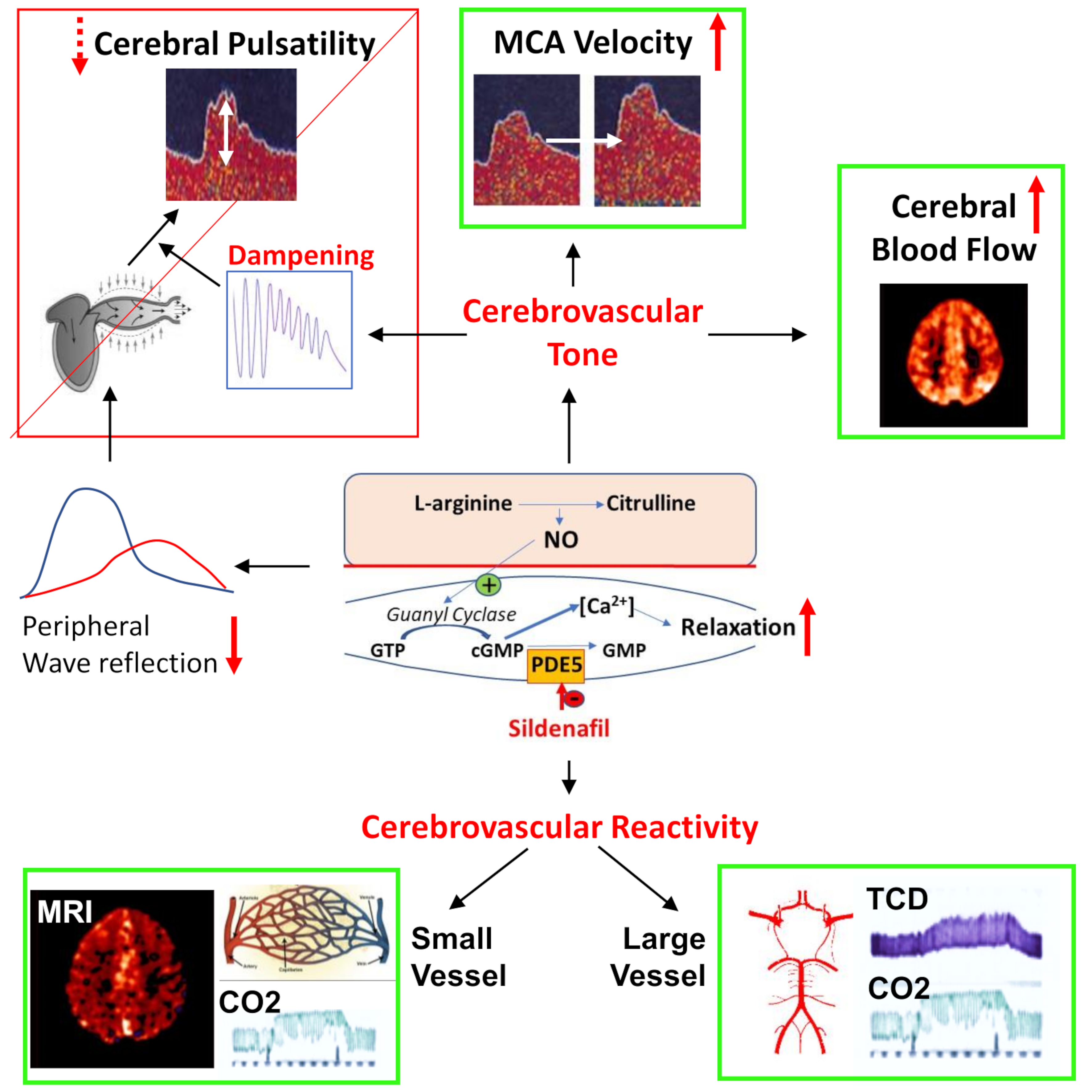

Supplement: 324327 Graphical Abstract [file EMS196246-supplement-324327_Graphical_Abstract.jpg]
