# Supplementary material for: Cerebrovascular Effects of Sildenafil in Small Vessel Disease: The OxHARP Trial
Source: Circ Res. Author manuscript; Available in PMC 2024 Jul 9. (PMC11227301; doi:10.1161/CIRCRESAHA.124.324327)
Supplement: 324327 Major Resources Table [file EMS196246-supplement-324327_Major_Resources_Table.pdf]

Major Resources Table

In order to allow validation and replication of experiments, all essential research materials listed in the Methods should be included in the Major Resources Table below. Authors are encouraged to use public repositories for protocols, data, code, and other materials and provide persistent identifiers and/or links to repositories when available. Authors may add or delete rows as needed.

Animals (in vivo studies)

Species	Vendor or Source	Background Strain	Sex	Persistent ID / URL
NA				

Genetically Modified Animals

	Species	Vendor or Source	Background Strain	Other Information	Persistent ID / URL
**Parent - Male**	NA				
**Parent - Female**	NA				

Antibodies

Target antigen	Vendor or Source	Catalog #	Working concentration	Lot # (preferred but not required)	Persistent ID / URL
NA					
NA					

DNA/cDNA Clones

Clone Name	Sequence	Source / Repository	Persistent ID / URL
NA			
NA			
NA			

Cultured Cells

Name	Vendor or Source	Sex (F, M, or unknown)	Persistent ID / URL
NA			
NA			
NA			

Data & Code Availability

Description	Source / Repository	Persistent ID / URL
Anonymised OxHARP trial dataset	Trial	NA – will be made available following primary publication

Other

Description	Source / Repository	Persistent ID / URL

DOI [to be added]

ARRIVE GUIDELINES

The ARRIVE guidelines (https://arriveguidelines.org/) are a checklist of recommendations to improve the reporting of research involving animals. Key elements of the study design should be included below to better enable readers to scrutinize the research adequately, evaluate its methodological rigor, and reproduce the methods or findings.

Study Design

**Groups**	**Sex**	**Age**	**Number (prior to experiment)**	**Number (after termination)**	**Littermates (Yes/No)**	**Other description**
Group 1 (Control)	NA					
Group 2						
Add more if needed						

Sample Size: Please explain how the sample size was decided Please provide details of any a *prior* sample size calculation, if done.

At a power level of 0.9, with a 2-sided significance of 5%, a clinically relevant 0.12 unit change in pulsatility index (equivalent to a ~20% difference in risk of recurrent stroke), and conservatively allowing for a standard deviation of differences in PI between repeated measures of 0.2, gives an estimated minimum sample size of 32 patients (paired t-test). Allowing for a 15% drop-out rate, 38 patients would be required. A sample size of 66 achieves 90% power to detect the non-inferiority of sildenafil c.f. cilostazol using a non-inferiority margin of 0.08 (and mean of paired differences 0) at α=0.025 (for a 95% CI) with a within-subject variance of 0.02 (equivalent to a SD of differences 0..[23,24] This equates to 75 patients in total with a 12% drop out rate.

Inclusion Criteria

- Participant is willing and able to give informed consent for participation in the study.
- Male or Female, aged 18 years or above.
- Can record MCA waveform on at least one side ('useable TCD window')
- Non-disabling, ischaemic stroke or probable TIA requiring treatment, >1 month prior to randomisation, of either cryptogenic or lacunar aetiology, confirmed clinically or on brain imaging
- White matter hyperintensities on MRI (Fazekas scale) or CT (Blennow scale) consistent with cerebral small vessel disease:
    - Age <60: MRI - Fazekas score 1 – 3 (max 2 points in periventricular or deep score)
      CT – Blennow score 1 – 3 (max 2 points in periventricular or deep score)
    - Age >60: MRI - Fazekas score 1 – 4 (max 2 points in periventricular or deep score)
      CT – Blennow score 1 – 4 (max 2 points in periventricular or deep score)

Exclusion Criteria

- Pregnant or breastfeeding women, women of childbearing age not taking contraception.
    - Acceptable contraception in women of childbearing age is a "highly effective" contraceptive measure as defined by the Clinical Trials Facilitation Group

DOI [to be added]

(http://www.hma.eu/fileadmin/dateien/Human_Medicines/01-About_HMA/Working_Groups/CTFG/2014_09_HMA_CTFG_Contraception.pdf) and includes combined (oestrogen and progesterone containing) or progesterone-only contraception associated with inhibition of ovulation, or intrauterine device or bilateral tubal occlusion

- Other major neurological or psychiatric conditions affecting the brain and interfering with the study design (e.g. multiple sclerosis)
- Other causes of stroke such as
  - ≥50% luminal stenosis (NASCET) in large arteries supplying the infarct area
  - major-risk cardioembolic source of embolism (permanent or paroxysmal atrial fibrillation, sustained atrial flutter, intracardiac thrombus, prosthetic cardiac valve, atrial myxoma or other cardiac tumours, mitral stenosis, recent (<4 weeks) myocardial infarction, left ventricular ejection fraction less than 30%, valvular vegetations, or infective endocarditis)
  - other specific causes of stroke (e.g. arteritis, dissection, drug misuse)
- Large vessel occlusion on MRA or CTA (carotid, basilar or MCA)
- Modified Rankin Score >3 (requires assistance to walk)
- Unable to swallow
- Renal impairment (eGFR <35ml/min)
- Significant biochemical abnormalities (sodium <130, K+ <2.5 or >5.5, LFTs >3 x upper limit of normal range)
- Life expectancy <2 years
- Contraindication to active agents
  - Concurrent use of alphablocker
  - Regular use of nitrate (ISMN, GTN, other)
  - Heart failure (NYHA 2-4)
  - Severe aortic stenosis
  - Bilateral renal artery stenosis
  - Uncontrolled arrhythmias
  - Previous priapism
  - Anatomical deformation of the penis
  - Recent myocardial infarction (within 6 months)
  - Unstable angina
  - History of non-arteritic ischaemic optic neuropathy
  - Hypotension: BP <90/60

DOI [to be added]

- Haemodynamically significant aortic / mitral valve disease
  - Sickle cell disease, myeloma, leukaemia
  - Uncontrolled hypertension (BP >180/110 despite treatment with 3 antihypertensives)
- Scheduled elective surgery or other procedures requiring general anaesthesia during the study.
- Any other significant disease or disorder which, in the opinion of the Investigator, may either put the participants at risk because of participation in the study or the participant's ability to participate in the study.
- Participants who have participated in another research study involving an investigational product in the past 12 weeks.
- Use of an anticoagulant (warfarin, dabigatran, rivaroxaban etc) or more than one antiplatelet drug.
- Predisposition to intracerebral haemorrhage (previous ICH, likely cerebral amyloid angiopathy) or intraocular haemorrhage (uncontrolled diabetic retinopathy or neovascularisation)
- Allergy to constituents of medications or components of placebo / overencapsulation
- Use of CYP induces that interact with study medications (ketoconazole, erythromycin).

Exclusion criteria specific for MRI substudy:

- Not able to transfer to MRI scanner
- Active respiratory illness (such as moderate to severe asthma or COPD) such that they are unable to tolerate MRI or unable to lie flat
- Claustrophobia
- Contraindication to MRI scan (pacemaker, aneurysm clip etc)

Other significant brain disorder that may confound interpretation of MRI imaging (multiple sclerosis, brain tumour)

Randomization

Randomisation will be performed by the Huddersfield PMU at the point of overencapsulation of medication in advance of provision of treatments to the Oxford Clinical Trial pharmacy. Study numbers will then be provided in sequence to participants at the Oxford Clinical Trial pharmacy. Each sequential study ID will be randomly allocated to one of six different treatment schedules, as outlined in the drug allocation table on page 11, to produce three groups of 12 and three groups of 13 patients. The randomisation schedule will be generated by Huddersfield PMU.

Blinding

The study will be blinded to the participant, blinded to the core study team and blinded to the endpoint assessors (TCD measurement and MRI analysis). Blinding will be maintained by overencapsulation of medication, and provision of dummy placebo at mid-day dosing during the cilostazol treatment phase.

DOI [to be added]
