# Supplementary material for: Cerebrovascular Effects of Sildenafil in Small Vessel Disease: The OxHARP Trial
Source: Circ Res. 2024 Jun 4;135(2):320–31. doi: 10.1161/CIRCRESAHA.124.324327 (PMC11227301; doi:10.1161/CIRCRESAHA.124.324327)

Supplementary Data

Cerebrovascular effects of sildenafil in small vessel disease: the OxHARP trial

Alastair JS Webb, Jacqueline S Birks, Karolina Feakins, Amy Lawson, Osian Llwyd PhD, Jesse Dawson, Alexander Rothman, David Werring, Catriona R Stewart, James Thomas

Inclusion Criteria

- Participant is willing and able to give informed consent for participation in the study.
- Male or Female, aged 18 years or above.
- Can record MCA waveform on at least one side ('useable TCD window')
- Non-disabling, ischaemic stroke or probable TIA requiring treatment, >1 month prior to randomisation, of either cryptogenic or lacunar aetiology, confirmed clinically or on brain imaging
- White matter hyperintensities on MRI (Fazekas scale) or CT (Blennow scale) consistent with cerebral small vessel disease:
    - Age <60: MRI - Fazekas score 1 – 3 (max 2 points in periventricular or deep score)
      CT – Blennow score 1 – 3 (max 2 points in periventricular or deep score)
    - Age >60: MRI - Fazekas score 1 – 4 (max 2 points in periventricular or deep score)
      CT – Blennow score 1 – 4 (max 2 points in periventricular or deep score)

Exclusion Criteria

- Pregnant or breastfeeding women, women of childbearing age not taking contraception.
    - Acceptable contraception in women of childbearing age is a "highly effective" contraceptive measure as defined by the Clinical Trials Facilitation Group (http://www.hma.eu/fileadmin/dateien/Human_Medicines/01-About_HMA/Working_Groups/CTFG/2014_09_HMA_CTFG_Contraception.pdf) and includes combined (oestrogen and progesterone containing) or progesterone-only contraception associated with inhibition of ovulation, or intrauterine device or bilateral tubal occlusion
- Other major neurological or psychiatric conditions affecting the brain and interfering with the study design (e.g. multiple sclerosis)
- Other causes of stroke such as
    - ≥50% luminal stenosis (NASCET) in large arteries supplying the infarct area
    - major-risk cardioembolic source of embolism (permanent or paroxysmal atrial fibrillation, sustained atrial flutter, intracardiac thrombus, prosthetic cardiac valve, atrial myxoma or other cardiac tumours, mitral stenosis, recent (<4 weeks) myocardial infarction, left ventricular ejection fraction less than 30%, valvular vegetations, or infective endocarditis)
    - other specific causes of stroke (e.g. arteritis, dissection, drug misuse)
- Large vessel occlusion on MRA or CTA (carotid, basilar or MCA)
- Modified Rankin Score >3 (requires assistance to walk)
- Unable to swallow
- Renal impairment (eGFR <35ml/min)
- Significant biochemical abnormalities (sodium <130, K+ <2.5 or >5.5, LFTs >3 x upper limit of normal range)
- Life expectancy <2 years
- Contraindication to active agents

- Concurrent use of alphablocker
- Regular use of nitrate (ISMN, GTN, other)
- Heart failure (NYHA 2-4)
- Severe aortic stenosis
- Bilateral renal artery stenosis
- Uncontrolled arrhythmias
- Previous priapism
- Anatomical deformation of the penis
- Recent myocardial infarction (within 6 months)
- Unstable angina
- History of non-arteritic ischaemic optic neuropathy
- Hypotension: BP <90/60
- Haemodynamically significant aortic / mitral valve disease
- Sickle cell disease, myeloma, leukaemia
- Uncontrolled hypertension (BP >180/110 despite treatment with 3 antihypertensives)

- Scheduled elective surgery or other procedures requiring general anaesthesia during the study.
- Any other significant disease or disorder which, in the opinion of the Investigator, may either put the participants at risk because of participation in the study or the participant's ability to participate in the study.
- Participants who have participated in another research study involving an investigational product in the past 12 weeks.
- Use of an anticoagulant (warfarin, dabigatran, rivaroxaban etc) or more than one antiplatelet drug.
- Predisposition to intracerebral haemorrhage (previous ICH, likely cerebral amyloid angiopathy) or intraocular haemorrhage (uncontrolled diabetic retinopathy or neovascularisation)
- Allergy to constituents of medications or components of placebo / overencapsulation
- Use of CYP induces that interact with study medications (ketoconazole, erythromycin).

Exclusion criteria specific for MRI substudy:

- Not able to transfer to MRI scanner
- Active respiratory illness (such as moderate to severe asthma or COPD) such that they are unable to tolerate MRI or unable to lie flat
- Claustrophobia
- Contraindication to MRI scan (pacemaker, aneurysm clip etc)
- Other significant brain disorder that may confound interpretation of MRI imaging (multiple sclerosis, brain tumour)

Supplemental Table 1. Clinical characteristics of population included in the OxHARP trial, subdivided by inclusion in study arms with only physiological testing, MRI imaging on sildenafil and placebo only or MRI imaging on all three interventions. P-values are determined from chi-squared tests and ANOVA for discrete and continuous variables respectively. TIA=transient ischaemic attack; WMH = white matter hyperintensities; MoCA = Montreal Cognitive Assessment.

Variables	2 MRIs	3 MRIs	Physiology Only	All participants	p-value
N	30	35*	10	75	
Age	70 (7.2)	68 (8)	72 (7.6)	70 (7.7)	0.31
Male	26 (86)	25 (71)	8 (80)	59 (78)	0.33
Diabetes	1 (3)	4 (11)	0 (0)	5 (6)	0.28
Hypertension	22 (73)	27 (77)	8 (80)	57 (76)	0.89
Smoker	4 (13)	4 (11)	6 (66)	8 (10)	0.58
White	29 (96)	35 (100)	10 (100)	74 (98)	0.47
Alcohol (units)	10 (5 - 20)	8 (0 - 20)	7 (1 - 14)	10 (1 - 20)	0.95
Event					
TIA	9 (30)	16 (45)	5 (50)	30 (40)	0.34
Stroke	21 (70)	19 (54)	5 (50)	45 (60)	
WMH severity					
Mild	16 (53)	18 (51)	6 (60)	40 (53)	0.60
Moderate	6 (20)	12 (34)	2 (20)	20 (26)	
Moderate-Severe	8 (26)	5 (14)	2 (20)	15 (20)	
Systolic BP	128 (18)	123 (16)	138 (18)	127 (18)	0.069
Diastolic BP	71 (9.6)	69 (10)	74 (11)	70 (10)	0.43
MoCA	27 (25.25 - 28)	27 (25 - 29)	26 (22.25 - 26)	27 (25 - 28)	0.11
Pulsatility Index	1.02 (0.18)	0.97 (0.14)	1.03 (0.12)	1.00 (0.16)	0.36
CVR-TCD	6.5 (2.3)	7.77 (2.2)	5.96 (1.7)	6.9 (2.3)	0.039
PSV	79.7 (15)	81.9 (19)	67.2 (12)	79.1 (17)	0.051
EDV	31.9 (8.2)	34 (8.9)	26 (3.8)	32.1 (8.4)	0.027

Supplemental Table 2. Clinical characteristics of population included in the OxHARP trial, subdivided by allocated randomisation order. TIA=transient ischaemic attack; MI = myocardial infarction.

Sequence	S – P - C	S – C - P	C – S - P	C – P - S	P – S - C	P – C - S	total
**N**	14	15	14	7	12	13	75
**Age**	71.3 (7.8)	69.4 (6.8)	68.1 (8.3)	65.4 (10.2)	72.4 (7.4)	69.7 (6.5)	69.7 (7.7)
**Male (%)**	10 (71)	10 (67)	12 (86)	6 (86)	8 (67)	13 (100)	59 (79%)
**Stroke**	7	10	9	6	5	8	45
**TIA**	7	5	5	1	7	5	30
**Diabetes (%)**	0 (0)	2 (13)	0 (0)	0 (0)	2 (20)	1 (8)	5 (7)
**Hypertension (%)**	12 (86)	11 (73)	11 (79)	3 (43)	10 (83)	10 (77)	57 (76)
**Hypercholesteriemia**	9 (64)	13 (75)	14 (100)	7 (100)	11 (92)	11 (85)	65 (87)
**MI**	0 (0)	1 (7)	0 (0)	1 (14)	0 (0)	1 (7)	3 (4)
**Current Smoker**	3	0	0	2	0	3	8
**Former Smoker**	7	9	10	4	5	5	40
**Never Smoked**	4	6	4	1	6	4	25
**e-cigarettes**	0	0	1	0	0	0	1
**Antihypertensives**	10	12	12	4	8	8	54
**Lipid lowering**	11	14	14	7	11	13	70
**No Antiplatelet**	2	0	1	0	0	0	3
**Aspirin**	3	1	1	0	3	2	10
**Clopidogrel**	9	14	12	7	9	11	62

Supplemental Table 3. Pairwise, within-individual differences in MRI indices between sildenafil, placebo and cilostazol by brain tissue type. Results are presented as mean difference, with 95% confidence intervals and p-values determined by mixed effect linear models, adjusted for age, gender, visit number and allocated randomisation order, for percentage cerebrovascular reactivity of BOLD MRI signal per mmHg change in end-tidal CO2 (CVR); to the estimated time delay between change in etCO2 and change in BOLD signal; arrival time of labelled blood flow on pcASL and estimated absolute cerebral perfusion, on ASL.

	CVR (% per mmHg)	p-val	CVR delay (s)	p-val	Perfusion (mls/100mg/min)	p-val	Arrival Time (s)	p-val
**Sildenafil vs Placebo**								
WMH	0.07 (0.00 , 0.14)	0.0432	-4.55 (-7.62 , -1.47)	0.0047	1.82 (0.5 , 3.15)	0.008	-0.01 (-0.07 , 0.04)	0.69
NAWM	0.06 (0.00 , 0.12)	0.0483	-3.58 (-6.09 , -1.07)	0.0063	2.12 (0.66 , 3.58)	0.006	-0.02 (-0.06 , 0.01)	0.2
Cortical Grey Matter	0.12 (-0.01 , 0.26)	0.07	-3.23 (-5.92 , -0.55)	0.02	3.69 (1.59 , 5.8)	0.001	-0.02 (-0.06 , 0.01)	0.21
Subcortical Grey Matter	0.15 (0.02 , 0.28)	0.0294	-2.84 (-5.59 , -0.08)	0.044	3.93 (1.62 , 6.25)	0.002	-0.02 (-0.07 , 0.04)	0.53
Brainstem	0.1 (-0.05 , 0.24)	0.19	-0.43 (-3.03 , 2.16)	0.74	2.64 (0.22 , 5.06)	0.033	-0.01 (-0.07 , 0.05)	0.79
**Cilostazol vs Placebo**								
WMH	0.06 (-0.15 , 0.28)	0.56	-3.51 (-7.55 , 0.54)	0.08	1.34 (-0.45 , 3.12)	0.13	0.02 (-0.04 , 0.07)	0.47
NAWM	0.04 (-0.1 , 0.18)	0.54	-4.22 (-6.87 , -1.56)	0.0042	1.66 (-0.16 , 3.49)	0.07	0.01 (-0.05 , 0.07)	0.82
Cortical Grey Matter	0.22 (-0.19 , 0.63)	0.26	-4.45 (-8.22 , -0.68)	0.0238	2.65 (-0.31 , 5.61)	0.08	0.02 (-0.03 , 0.07)	0.45
Subcortical Grey Matter	0.19 (-0.12 , 0.49)	0.21	-3.72 (-6.79 , -0.64)	0.0214	2.38 (-0.28 , 5.04)	0.08	0.02 (-0.05 , 0.08)	0.59
Brainstem	0.17 (-0.24 , 0.58)	0.38	-2.52 (-5.02 , -0.01)	0.0491	1.18 (-1.73 , 4.09)	0.4	0.01 (-0.1 , 0.11)	0.88
**Sildenafil vs Cilostazol**								
WMH	0.01 (-0.19 , 0.22)	0.91	0.25 (-4.74 , 5.23)	0.92	0.21 (-1.1 , 1.52)	0.74	0.02 (-0.05 , 0.09)	0.56
NAWM	0.03 (-0.14 , 0.2)	0.74	0.22 (-4.17 , 4.62)	0.91	0.57 (-1.39 , 2.53)	0.55	0.01 (-0.07 , 0.08)	0.81
Cortical Grey Matter	-0.06 (-0.41 , 0.29)	0.71	1.72 (-1.7 , 5.13)	0.92	-0.32 (-2.57 , 1.94)	0.77	-0.02 (-0.08 , 0.05)	0.58
Subcortical Grey Matter	0.05 (-0.31 , 0.4)	0.79	1.57 (-2.22 , 5.36)	0.91	0.32 (-2.73 , 3.37)	0.83	0 (-0.07 , 0.08)	0.94
Brainstem	-0.02 (-0.42 , 0.38)	0.93	2.92 (-1.34 , 7.17)	0.56	0.13 (-3.42 , 3.69)	0.94	-0.02 (-0.11 , 0.08)	0.72

Supplemental Table 4. Analysis of the potential mediating effects of sildenafil versus placebo on systemic haemodynamics for cerebrovascular outcomes. Effects of sildenafil versus placebo on the outcome variable are decomposed into direct effects versus indirect mediated by the mediator. Two way decompositions with mediation and interaction effects were also performed, but there was only a significant mediator*treatment interaction for aortic mean BP to cerebral perfusion measures, where mediation effects were only significant in the treatment group (indicated by *). TCD=transcranial doppler ultrasound; PI = pulsatility index; PWV = pulse wave velocity; BP = blood pressure; MFV = mean flow velocity; WMH = white matter hyperintensities; NAWM = normal appearing white matter; CVR = cerebrovascular reactivity.

**Model**						
Mediator	Outcome	**Direct Effect**	**p-value**	**Indirect Effect**	**p-value**	**% Mediated**
Cerebral Blood Flow on TCD						
Aortic PI	Cerebral PI	0.02	0.21	0.001	0.95	1
Aortic mean BP	Cerebral PI	0.02	0.21	0.001	0.95	4
Aortic PWV	Cerebral PI	0.02	0.18	-0.0004	0.90	0
Aortic mean BP	Cerebral MFV	2.96	0.004	0.36	0.45	11
Aortic PWV	Cerebral MFV	3.31	<0.001	0.02	0.88	0
Cerebral Perfusion on MRI						
Aortic mean BP	WMH	1.41	0.036	0.21	0.48*	13
Aortic mean BP	NAWM	1.80	0.04	0.56	0.10*	23
Aortic mean BP	Grey Matter	1.84	0.05	0.55	0.10*	22
Cerebral MFV	WMH	0.91	0.15	0.71	0.01	44
Cerebral MFV	NAWM	1.65	0.03	0.73	0.006	31
Cerebral MFV	Grey Matter	1.63	0.03	0.75	<0.001	31
Cerebrovascular Reactivity on MRI						
CVR-TCD	CVR-WMH	0.026	0.55	0.027	0.06	40
CVR-TCD	CVR-NAWM	0.02	0.46	0.01	0.046	26
CVR-TCD	CVR-GM	0.10	0.25	0.03	0.06	20

Supplemental Figure 1. Within-individual differences in transcranial ultrasound measures. The individual differences are shown, with summary mean values in each index with 95% confidence intervals for middle cerebral artery pulsatility (MCA-PI), cerebrovascular reactivity from the beta-coefficient from a linear model for the association between end-tidal CO2 and mean flow velocity (CVR), mean MCA flow velocity (MFV) and the cerebrovascular conductance index (CVCi), calculated by MFV divided by mean aortic blood pressure. Differences are shown for sildenafil versus placebo (S-P), sildenafil versus cilostazol (S-C) and cilostazol versus placebo (C-P).

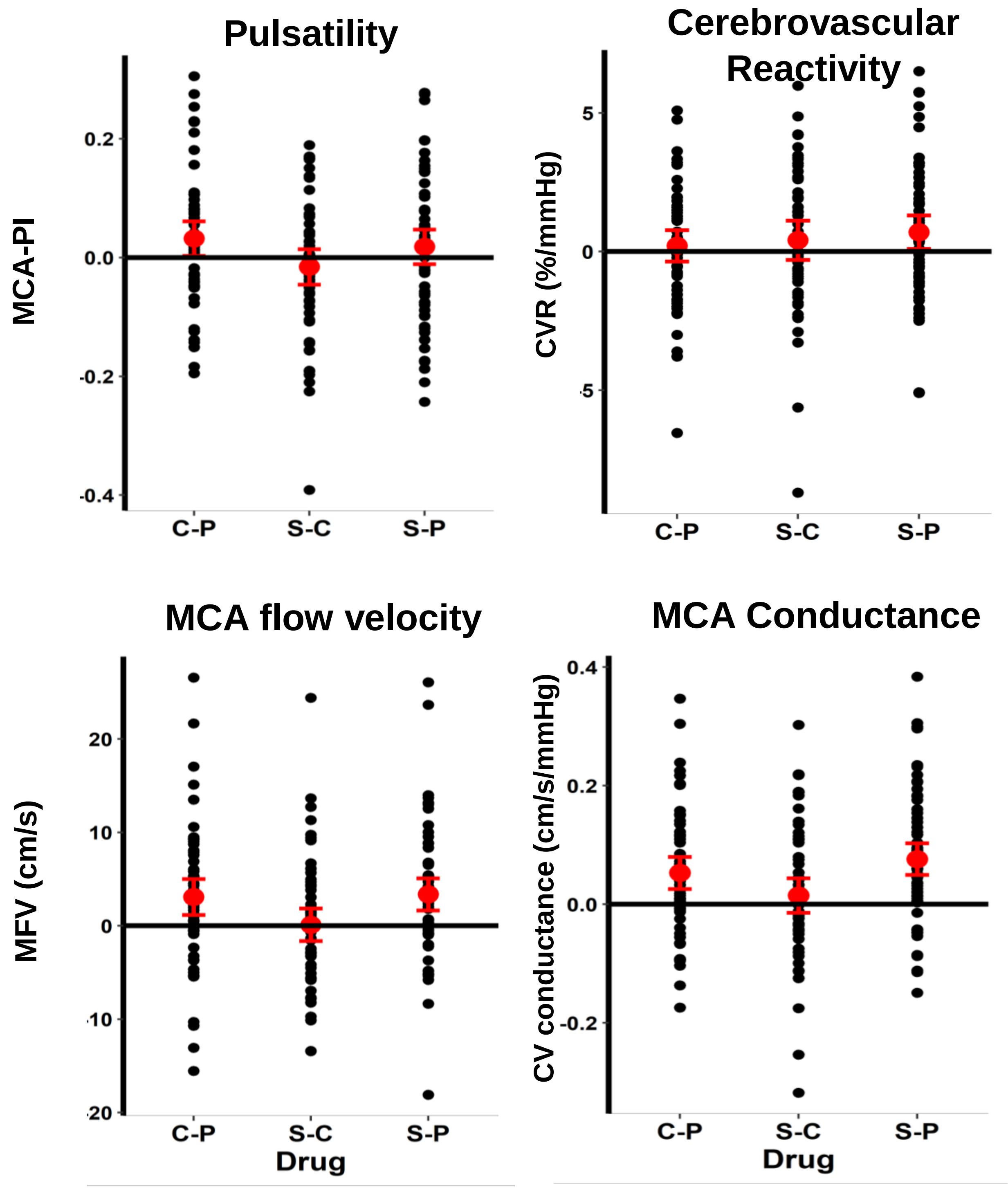

Supplemental Figure 2. Mean within-individual differences in transcranial ultrasound and aortic blood pressure measures, for systolic and diastolic blood flow. The mean, within-individual difference in each index is shown with 95% confidence intervals for peak systolic velocity (PSV), end-diastolic velocity (EDV) and aortic systolic (SBP) and diastolic (DBP) blood pressures. Differences are shown for sildenafil versus placebo (S-P), sildenafil versus cilostazol (S-C) and cilostazol versus placebo (C-P).4

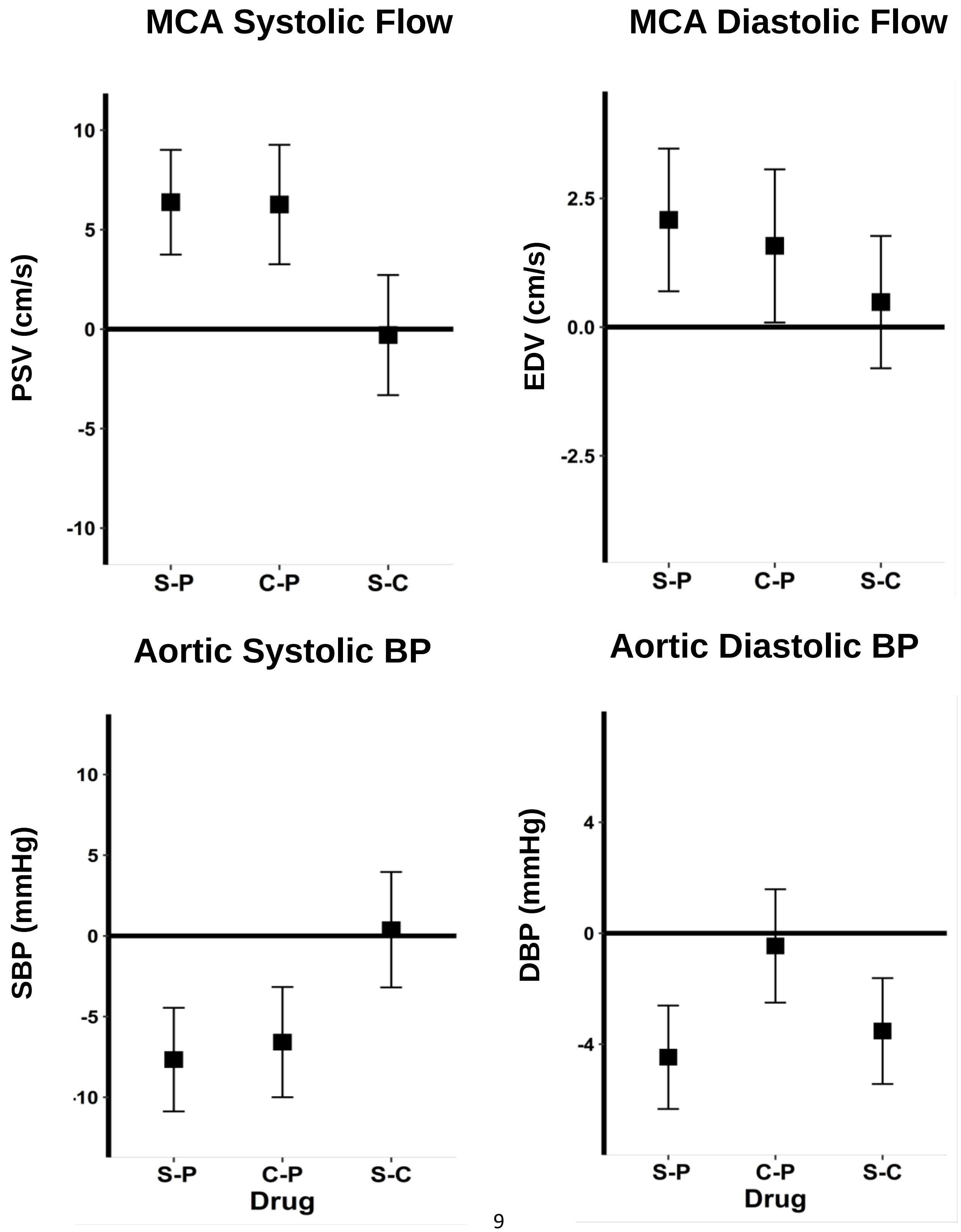

Supplemental Figure 3. Regional differences between cilostazol and placebo for the primary imaging outcomes. The mean, within-individual difference on MRI imaging for cilostazol minus placebo values is shown on a voxel-wise basis, following registration to standard MNI space and Gaussian smoothing. Results are shown for A) CVR: cerebrovascular reactivity, in percentage change in BOLD signal per mmHg end-tidal CO2); B) CVR response: difference in the delay between change in etCO2 and change in BOLD signal during CVR in seconds; C) Difference in perfusion on ASL, in ml/min/100g; D) Difference in arrival time of blood flow during ASL, in seconds

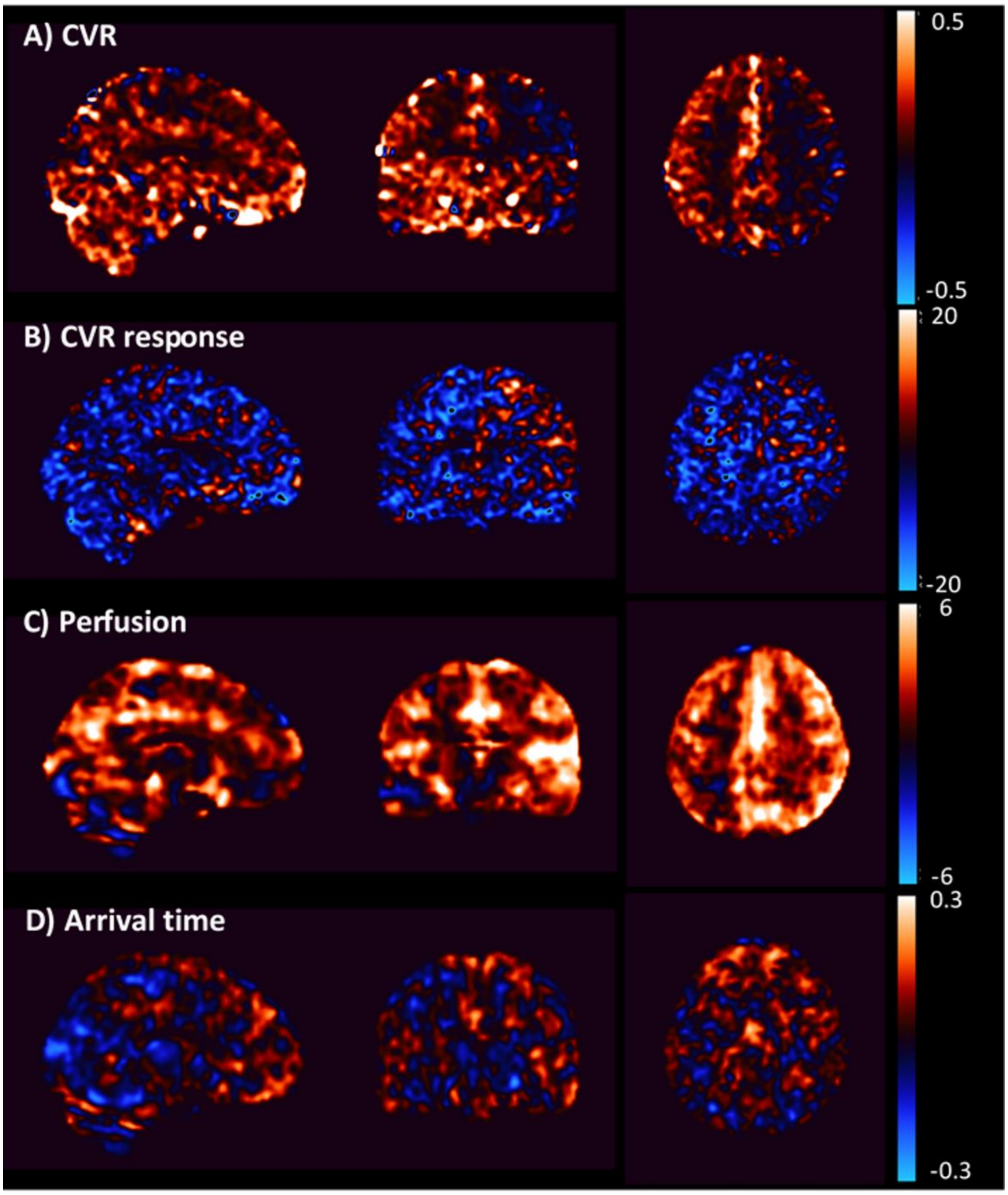

Supplemental Figure 4. Regional difference between sildenafil and cilostazol for the primary imaging outcomes. The mean, within-individual difference on MRI imaging for sildenafil minus cilostazol values is shown on a voxel-wise basis, following registration to standard MNI space and Gaussian smoothing. Results are shown for A) CVR: cerebrovascular reactivity, in percentage change in BOLD signal per mmHg end-tidal CO2); B) CVR response: difference in the delay between change in etCO2 and change in BOLD signal during CVR in seconds; C) Difference in perfusion on ASL, in ml/min/100g; D) Difference in arrival time of blood flow during ASL, in seconds

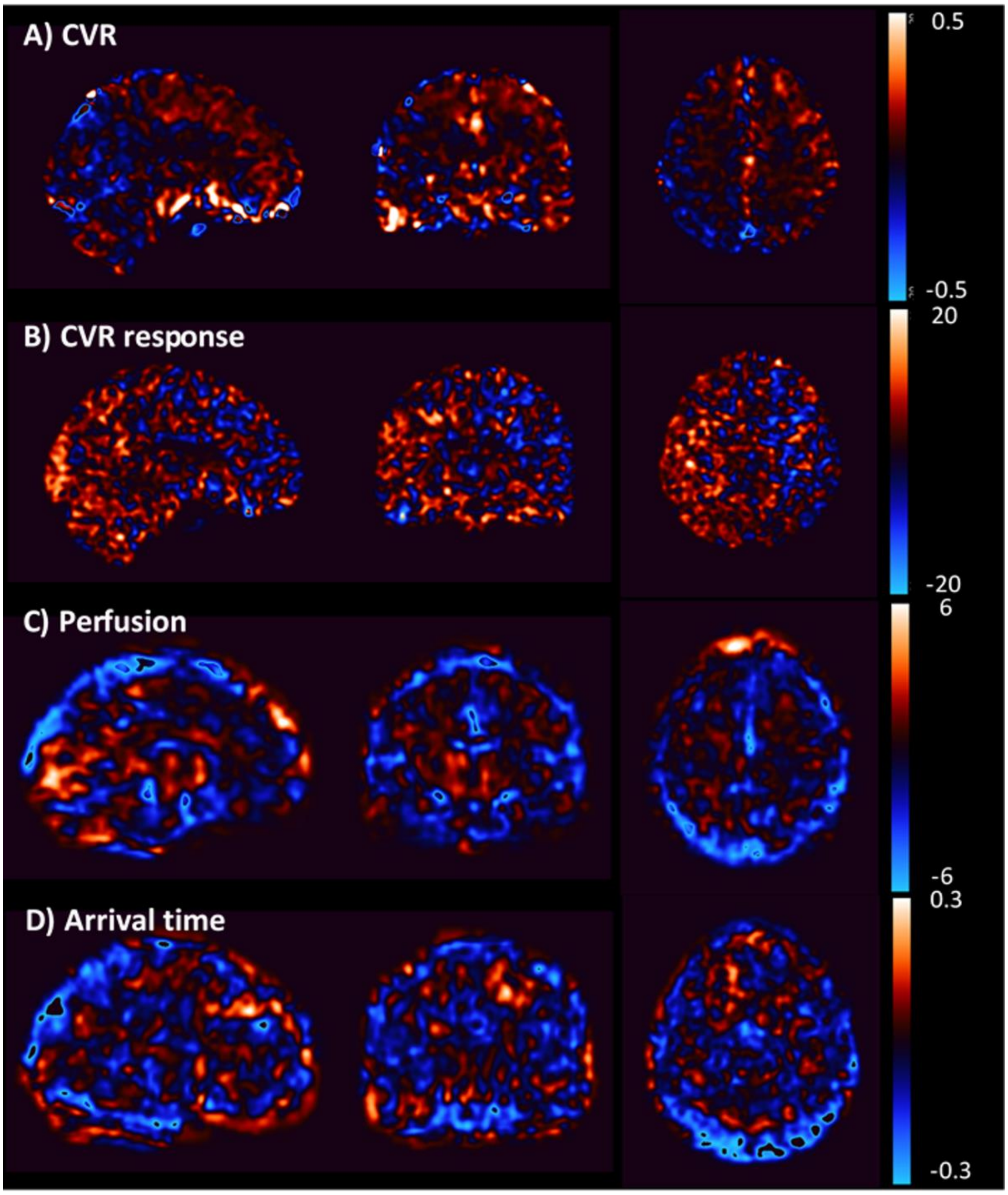

Supplement: Supplementary file 1 [file res-135-320-s001.pdf]
